# BCS thermal vacuum of fermionic superfluids and its perturbation theory

**DOI:** 10.1038/s41598-018-30438-1

**Published:** 2018-08-10

**Authors:** Xu-Yang Hou, Ziwen Huang, Hao Guo, Yan He, Chih-Chun Chien

**Affiliations:** 1 Department of Physics, Southeast University, Jiulonghu Campus, Nanjing, 211189 China; 2 College of Physical Science and Technology, Sichuan University, Chengdu, Sichuan 610064 China; 3 School of Natural Sciences, University of California, Merced, CA 95343 USA; 4 Present Address: Department of Physics and Astronomy, Northwestern University, Evanston, IL 60208 USA

## Abstract

The thermal field theory is applied to fermionic superfluids by doubling the degrees of freedom of the BCS theory. We construct the two-mode states and the corresponding Bogoliubov transformation to obtain the BCS thermal vacuum. The expectation values with respect to the BCS thermal vacuum produce the statistical average of the thermodynamic quantities. The BCS thermal vacuum allows a quantum-mechanical perturbation theory with the BCS theory serving as the unperturbed state. We evaluate the leading-order corrections to the order parameter and other physical quantities from the perturbation theory. A direct evaluation of the pairing correlation as a function of temperature shows the pseudogap phenomenon, where the pairing persists when the order parameter vanishes, emerges from the perturbation theory. The correspondence between the thermal vacuum and purification of the density matrix allows a unitary transformation, and we found the geometric phase associated with the transformation in the parameter space.

## Introduction

Quantum many-body systems can be described by quantum field theories^1–4^. Some available frameworks for systems at finite temperatures include the Matsubara formalism using the imaginary time for equilibrium systems^1,5^ and the Keldysh formalism of time-contour path integrals^3,6^ for non-equilibrium systems. There are also alternative formalisms. For instance, the thermal field theory^7–9^ is built on the concept of thermal vacuum. The idea of thermal vacuum is to construct temperature-dependent augmented states and rewrite the statistical average of observables as quantum-mechanical expectation values. Thermal field theory was introduced a while ago^7,10^, and more recently it has found applications beyond high-energy physics^9^.

The thermal vacuum of a non-interacting bosonic or fermionic system has been obtained by a Bogoliubov transformation of the corresponding two-mode vacuum^11^, where an auxiliary system, called the tilde system, is introduced to satisfy the statistical weight. The concepts of Bogoliubov transformation and unitary inequivalent representations are closely related to the development of thermal vacuum theory^9^, and they are also related to the quantum Hall effect^12^ and orthogonality catastrophe^4^. The thermal vacuum of an interacting system can be constructed if a Bogoliubov transformation of the corresponding two-mode vacuum is found. In the following, we will use the BCS theory of fermionic superfluids as a concrete example. By construction, the thermal vacuum provides an alternative interpretation of the statistical average. Nevertheless, we will show that the introduction of the thermal vacuum simplifies certain calculations from the level of quantum field theory to the level of quantum mechanics. As an example, we will apply the thermal field theory to develop a perturbation theory where the BCS thermal vacuum serves as the unperturbed state, and the fermion-fermion interaction ignored in the BCS approximation is the perturbation. Because the particle-hole channel, or the induced interaction^13^, is not included in the BCS theory, the BCS thermal vacuum inherits the same property and its perturbation theory does not produce the Gorkov-Melik-Barkhudarov effect^14^ predicting the transition temperature is more than halved.

The thermal vacuum of a given Hamiltonian can alternatively be viewed as a purification of the density matrix of the corresponding mixed state^15,16^. Since the thermal vacuum is a pure state, the statistical average of a physical quantity at finite temperatures is the expectation value obtained in the quantum mechanical manner. Therefore, one can find some physical quantities not easily evaluated in conventional methods. This is the reason behind the perturbation theory using the BCS thermal vacuum as the unperturbed state. The corrections from the full fermion-fermion interaction can be evaluated order-by-order following the standard time-independent quantum-mechanical perturbation theory^17^. In principle, the corrections to any physical quantities can be expressed as a perturbation series. In this work, the first-order corrections to the order parameter will be evaluated and analyzed.

Furthermore, the BCS thermal vacuum allows us to gain insights into the BCS superfluids. We will illustrate the applications of the BCS thermal vacuum by calculating the pairing correlation, which may be viewed as the quantum correlation matrix^18^ of the pairing operator. We found that the corrected order parameter vanishes at a lower temperature compared to the unperturbed one, and the pairing correlation persists above the corrected transition temperature. Thus, the perturbation theory offers an algebraic foundation for the pseudogap effect^19,20^, where the pairing effect persists above the transition temperature. Moreover, the purification of the density matrix of a mixed state allows a unitary transformation^15,16^. For the BCS thermal vacuum, this translates to a *U*(1) phase in its construction. By evaluating the analogue of the Berry phase^21^ along the *U*(1)-manifold of the unitary transformation, we found a thermal phase characterizing the thermal excitations of the BCS superfluid.

Throughout this paper, we choose *ℏ* = *k*_*B*_ = 1 and set |*q*_*e*_| = 1, where *q*_*e*_ is the electron charge. In quantum statistics, the expectation value of an operator ${\mathscr{A}}$ in a canonical ensemble is evaluated by the statistical (thermal) average

1 $${\langle {\mathscr{A}}\rangle }_{\beta }\equiv \frac{1}{Z}\,{\rm{Tr}}({{\rm{e}}}^{-\beta H}{\mathscr{A}}),$$

where *Z* = Tre^−*βH*^ is the partition function with the Hamiltonian *H* at temperature $T\equiv \frac{1}{\beta }$. A connection between the statistical average and quantum field theory is established by rewriting the partition function in the path-integral form and introducing the imaginary time *τ* = *it*. This method allows a correspondence between the partition functions in statistical mechanics and quantum field theory^3,4^. Therefore, one usually focuses on equilibrium statistical mechanics^2^, where the Matsubara frequencies are introduced^5^.

The central idea of thermal field theory is to express the statistical average over a set of mixed quantum states as the expectation value of a temperature-dependent pure state, called the thermal vacuum |0(*β*)〉^9,22^. Explicitly,

2 $${\langle {\mathscr{A}}\rangle }_{\beta }=\langle 0(\beta )|{\mathscr{A}}|0(\beta )\rangle ={Z}^{-1}\,\sum _{n}\,\langle n|{\mathscr{A}}|n\rangle {{\rm{e}}}^{-\beta {E}_{n}}.$$

Here |*n*〉 are the orthonormal eigenstates of the Hamiltonian *H* satisfying 〈*n*|*m*〉 = *δ*_*nm*_ with eigenvalues *E*_*n*_. Following refs^7,22^, one way to achieve this is to double the degrees of freedom of the system by introducing an auxiliary system identical to the one we are studying. This auxiliary system is usually denoted by the tilde symbol, so its Hamiltonian is $\tilde{H}$ and its eigenstates $|\tilde{n}\rangle$ satisfy $\tilde{H}|\tilde{n}\rangle ={E}_{n}|\tilde{n}\rangle$ and $\langle \tilde{n}|\tilde{m}\rangle ={\delta }_{nm}$.

It is imposed that th non-tilde bosonic (fermionic) operators commute (anti-commute) with their tilde counterparts^7,22^. To find |0(*β*)〉, one needs to consider the space spanned by the direct product of the tilde and non-tilde state-vectors $|n,\tilde{m}\rangle \equiv |n\rangle \otimes |\tilde{m}\rangle$. Hence, the thermal vacuum can be expressed as

3 $$|0(\beta )\rangle =\sum _{n}\,|n\rangle \otimes |\,{f}_{n}(\beta )\rangle =\sum _{n}\,{f}_{n}(\beta )|n,\tilde{n}\rangle ,$$

where $|\,{f}_{n}(\beta )\rangle ={f}_{n}(\beta )|\tilde{n}\rangle$ with ${f}_{n}^{\ast }(\beta ){f}_{m}(\beta )={Z}^{-1}\,{{\rm{e}}}^{-\beta {E}_{n}}{\delta }_{nm}$. It is also required that the operator ${\mathscr{A}}$, whose expectation values we are interested in, only acts on the non-tilde space^7,22^. Then, $\langle 0(\beta )|A|0(\beta )\rangle ={\sum }_{n}\,|\,{f}_{n}(\beta ){|}^{2}\langle n|{\mathscr{A}}|n\rangle$. Eq. (2) is satisfied if

4 $$|\,{f}_{n}(\beta ){|}^{2}={Z}^{-1}\,{{\rm{e}}}^{-\beta {E}_{n}}.$$

Therefore, the thermal vacuum can be expressed as

5 $$|0(\beta )\rangle =\sum _{n}\,\frac{1}{\sqrt{Z}}{{\rm{e}}}^{-\beta \frac{{E}_{n}}{2}+i{\chi }_{n}}|n,\tilde{n}\rangle ,$$

where we introduce a factor exp(*iχ*_*n*_) to each coefficient *f*_*n*_(*β*). This is allowed because one may view the thermal vacuum as a purification of the density matrix, and different purified states of the same density matrix can be connected by a unitary transformation^15,16^.

The thermal vacuum is usually not the ground state of either *H* or $\tilde{H}$, but it is the zero-energy eigenstate of $\hat{H}\equiv H-\tilde{H}$. Since the energy spectrum of $\hat{H}$ has no lower bound, it is not physical. In noninteracting bosons or fermions and the BCS superfluid, the thermal vacuum can be constructed by performing a unitary transformation *U*(*β*) on the two-mode ground state, $|0(\beta )\rangle =U(\beta )|0,\tilde{0}\rangle$ via a Bogoliubov transformation. In those cases, the thermal vacuum is an eigenstate of the “thermal Hamiltonian” *H*(*T*) ≡ *U*(*β*)*HU*^−1^(*β*) with eigenvalue *E*_0_, which is also the ground-state energy of *H*. This is because $H(T)|0(\beta )\rangle =UH|0,\tilde{0}\rangle ={E}_{0}U|0,\tilde{0}\rangle ={E}_{0}|0(\beta )\rangle$. Importantly, |0(*β*)〉 is also the ground state of *H*(*T*) since unitary transformations do not change the eigenvalues of an operator. The origin of the name “thermal vacuum” comes from the fact that it is the finite-temperature generalization of the two-mode ground state^7,8^. We remark that *U*(*β*) may contain creation and annihilation operators and may not be unitary in generic interacting systems. Based on the BCS thermal vacuum, we will develop a perturbation theory for systems where *U*(*β*) cannot be constructed explicitly.

## Results

### BCS Thermal Vacuum

We first give a brief review of the BCS theory. The fermion field operators *ψ*_*σ*_ and ${\psi }_{\sigma }^{\dagger }$ satisfy

6 $$\{{\psi }_{{\bf{k}}\sigma },{\psi }_{{\bf{k}}{\boldsymbol{^{\prime} }}\sigma ^{\prime} }^{\dagger }\}={\delta }_{{\bf{k}}{\bf{k}}{\boldsymbol{^{\prime} }}}{\delta }_{\sigma \sigma ^{\prime} },\,\sigma ,\sigma ^{\prime} =\uparrow \,,\,\downarrow $$

and all other anti-commutators vanish. The Hamiltonian of a two-component Fermi gas with attractive contact interactions is given by

7 $$H=\sum _{{\bf{k}}}\,{\psi }_{{\bf{k}}\sigma }^{\dagger }(\frac{{{\bf{k}}}^{2}}{2m}-\mu ){\psi }_{{\bf{k}}\sigma }-g\,\sum _{{\bf{k}}{\bf{p}}{\bf{q}}}\,{\psi }_{{\bf{k}}-{\bf{p}}\uparrow }^{\dagger }{\psi }_{{\bf{p}}\downarrow }^{\dagger }{\psi }_{{\bf{k}}-{\bf{q}}\downarrow }{\psi }_{{\bf{q}}\uparrow },$$

where *m* and *μ* are the fermion mass and chemical potential, respectively, *g* is the coupling constant, and ${\sum }_{{\bf{k}}}={\mathscr{V}}\,\int \,\frac{{d}^{3}{\bf{k}}}{{(2\pi )}^{3}}$ with ${\mathscr{V}}$ being the volume of the system. In the BCS theory, the pairing field leads to the gap function ${\rm{\Delta }}({\bf{x}})=g\langle {\psi }_{\uparrow }({\bf{x}}){\psi }_{\downarrow }({\bf{x}})\rangle$, which is also the order parameter in the broken-symmetry phase. Physically, pairing between fermions in the time-reversal states $({\bf{k}}\,\uparrow \,)$ and ($-{\bf{k}}\downarrow$) can make the Fermi sea unstable if the inter-particle interaction is attractive^23^.

If one takes only the **k** = 0 contribution in Eq. (7), the Hamiltonian of a homogeneous system can be approximated by the BCS form^24,25^ with ${\rm{\Delta }}=g\,{\sum }_{{\bf{p}}}\,\langle {\psi }_{-{\bf{p}}\downarrow }{\psi }_{{\bf{p}}\uparrow }\rangle$. Explicitly,

8 $${H}_{{\rm{BCS}}}=\frac{|{\rm{\Delta }}{|}^{2}}{g}+\sum _{{\bf{k}}\sigma }\,{\psi }_{{\bf{k}}\sigma }^{\dagger }(\frac{{{\bf{k}}}^{2}}{2m}-\mu ){\psi }_{{\bf{k}}\sigma }-\sum _{{\bf{k}}}\,({{\rm{\Delta }}}^{\ast }{\psi }_{{\bf{k}}\downarrow }{\psi }_{-{\bf{k}}\uparrow }+{\rm{\Delta }}{\psi }_{-{\bf{k}}\uparrow }^{\dagger }{\psi }_{{\bf{k}}\downarrow }^{\dagger }),$$

which can be diagonalized as

9 $${H}_{{\rm{BCS}}}=\sum _{{\bf{k}}}\,{E}_{{\bf{k}}}({\alpha }_{{\bf{k}}}^{\dagger }{\alpha }_{{\bf{k}}}+{\beta }_{-{\bf{k}}}^{\dagger }{\beta }_{-{\bf{k}}})+\sum _{{\bf{k}}}\,({\xi }_{{\bf{k}}}-{E}_{{\bf{k}}})+\frac{|{\rm{\Delta }}{|}^{2}}{g}.$$

This is achieved by implementing the Bogoliubov transformation, which can also be cast in the form of a similarity transformation:

10 $${\alpha }_{{\bf{k}}}={{\rm{e}}}^{G}{\psi }_{{\bf{k}}\uparrow }{{\rm{e}}}^{-G}=\,\cos \,{\varphi }_{{\bf{k}}}{\psi }_{{\bf{k}}\uparrow }-\,\sin \,{\varphi }_{{\bf{k}}}{\psi }_{-{\bf{k}}\downarrow }^{\dagger },\,{\beta }_{-{\bf{k}}}^{\dagger }={{\rm{e}}}^{G}{\psi }_{-{\bf{k}}\downarrow }{{\rm{e}}}^{-G}=\,\sin \,{\varphi }_{{\bf{k}}}{\psi }_{{\bf{k}}\uparrow }+\,\cos \,{\varphi }_{{\bf{k}}}{\psi }_{-{\bf{k}}\downarrow }^{\dagger }.$$

Here $\cos (2{\varphi }_{{\bf{k}}})=\frac{{\xi }_{{\bf{k}}}}{{E}_{{\bf{k}}}}$, $\sin (2{\varphi }_{{\bf{k}}})=\frac{|{\rm{\Delta }}|}{{E}_{{\bf{k}}}}$, ${E}_{{\bf{k}}}=\sqrt{{\xi }_{{\bf{k}}}^{2}+|{\rm{\Delta }}{|}^{2}}$ is the quasi-particle dispersion, $G={\sum }_{{\bf{k}}}\,{\varphi }_{{\bf{k}}}({\psi }_{{\bf{k}}\uparrow }{\psi }_{-{\bf{k}}\downarrow }+$ ${\psi }_{{\bf{k}}\uparrow }^{\dagger }{\psi }_{-{\bf{k}}\downarrow }^{\dagger })$ is the generator of the transformation. Since ${G}^{\dagger }=-\,G$, *e*^−*G*^ is unitary. The field operators of the quasi-particles satisfy the anti-commutation relations

11 $$\{{\alpha }_{{\bf{k}}},{\alpha }_{{\bf{k}}{\boldsymbol{^{\prime} }}}^{\dagger }\}={\delta }_{{\bf{k}}{\bf{k}}{\boldsymbol{^{\prime} }}},\{{\beta }_{{\bf{k}}},{\beta }_{{\bf{k}}{\boldsymbol{^{\prime} }}}^{\dagger }\}={\delta }_{{\bf{k}}{\bf{k}}{\boldsymbol{^{\prime} }}},$$

and all other anti-commutators vanish. We remark that the BCS theory only considers the particle-particle channel (pairing) contribution. As pointed out in ref.^26^, the BCS theory is not compatible with a split, density contribution to the chemical potential. It is, however, possible to add the particle-hole (density) diagrams to the Feynman diagrams describing the scattering process^13^ and obtain a modified effective interaction, which then leads to the Gorkov-Melik-Barkhudarov effect^14^ of suppressed superfluid transition temperature. Here we base the theory on the BCS theory, so the resulting thermal field theory also does not explicitly exhibit the particle-hole channel effects.

Rewriting the Bogoliubov transformation as a similarity transformation leads to a connection between the Fock-space vacuum |0〉 of the *ψ*_*σ*_ quanta and the BCS ground state |*g*〉, which can be viewed as the vacuum of the *α*_**k**_ and *β*_−**k**_ quasi-particles because *α*_**k**_|*g*〉 = 0 = *β*_−**k**_|*g*〉. Explicitly,

12 $$|g\rangle ={{\rm{e}}}^{G}|0\rangle =\prod _{{\bf{k}}}\,(\cos \,{\varphi }_{{\bf{k}}}+\,\sin \,{\varphi }_{{\bf{k}}}{\psi }_{{\bf{k}}\uparrow }^{\dagger }{\psi }_{-{\bf{k}}\downarrow }^{\dagger })|0\rangle .$$

We remark the relation between the similarity transformation of the fields and the unitary transformation of the Fock-space vacuum resembles the connection between the Schrodinger picture and the Heisenberg picture in quantum dynamics (see ref.^17^ for example).

The thermal vacuum of the BCS theory is constructed by introducing the tilde partners of the *α*_**k**_ and *β*_−**k**_ quanta, ${\tilde{\alpha }}_{{\bf{k}}}$ and ${\tilde{\beta }}_{-{\bf{k}}}$. They satisfy the algebra

13 $$\{{\tilde{\alpha }}_{{\bf{k}}},{\tilde{\alpha }}_{{\bf{k}}{\boldsymbol{^{\prime} }}}^{\dagger }\}={\delta }_{{\bf{k}}{\bf{k}}{\boldsymbol{^{\prime} }}},\{{\tilde{\beta }}_{{\bf{k}}},{\tilde{\beta }}_{{\bf{k}}{\boldsymbol{^{\prime} }}}^{\dagger }\}={\delta }_{{\bf{k}}{\bf{k}}{\boldsymbol{^{\prime} }}},$$

and all other anti-commutators vanish. Moreover, the tilde fields anti-commute with the quasi-particle quanta *α*_**k**_ and *β*_**k**_. Next, the two-mode BCS ground state is constructed as follows.

14 $$|g,\tilde{g}\rangle =\prod _{{\bf{k}}}\,|0,\tilde{0}{\rangle }_{{\alpha }_{{\bf{k}}}}\otimes |0,\tilde{0}{\rangle }_{{\beta }_{-{\bf{k}}}}.$$

Here $|0,\tilde{0}{\rangle }_{{\alpha }_{{\bf{k}}}}$ $(|0,\tilde{0}{\rangle }_{{\beta }_{-{\bf{k}}}})$ denotes the Fock-space vacuum of *α*_**k**_ and ${\tilde{\alpha }}_{{\bf{k}}}$ (*β*_−**k**_ and ${\tilde{\beta }}_{-{\bf{k}}}$).

The occupation number of each fermion quasi-particle state can only be 0 or 1. According to Eq. (3), the two-mode BCS thermal vacuum can be expressed as

15 $$|0(\beta )\rangle =\prod _{{\bf{k}}}\,\otimes {({f}_{0{\bf{k}}}|0,\tilde{0}\rangle }_{{\alpha }_{{\bf{k}}}}+{f}_{1{\bf{k}}}|1,\tilde{1}{\rangle }_{{\alpha }_{{\bf{k}}}})\otimes {({f}_{0{\bf{k}}}|0,\tilde{0}\rangle }_{{\beta }_{-{\bf{k}}}}+{f}_{1{\bf{k}}}|1,\tilde{1}{\rangle }_{{\beta }_{-{\bf{k}}}}),$$

where $|1,\tilde{1}{\rangle }_{{\alpha }_{{\bf{k}}}}={\alpha }_{{\bf{k}}}^{\dagger }{\tilde{\alpha }}_{{\bf{k}}}^{\dagger }|g,\tilde{g}\rangle$ and $|1,\tilde{1}{\rangle }_{{\beta }_{-{\bf{k}}}}={\beta }_{-{\bf{k}}}^{\dagger }{\tilde{\beta }}_{-{\bf{k}}}^{\dagger }|g,\tilde{g}\rangle$. The coefficients *f*_0**k**_ and *f*_1**k**_ can be deduced from Eq. (4). For each **k**, we define ${Z}_{{\bf{k}}}=1+{{\rm{e}}}^{-\beta {E}_{{\bf{k}}}}$ and then the partition function is $Z={\prod }_{{\bf{k}}}\,{Z}_{{\bf{k}}}$. Comparing with Eq. (4), we get

16 $$|\,{f}_{0{\bf{k}}}|={Z}_{{\bf{k}}}^{-\frac{1}{2}}=\frac{1}{\sqrt{1+{e}^{-\beta {E}_{{\bf{k}}}}}},\,|\,{f}_{1{\bf{k}}}|={Z}_{{\bf{k}}}^{-\frac{1}{2}}{{\rm{e}}}^{-\beta {E}_{{\bf{k}}}/2}=\frac{1}{\sqrt{1+{e}^{\beta {E}_{{\bf{k}}}}}}.$$

According to Eq. (5), one may choose a relative phase between the different two-mode states. Here, we choose *χ*_0_ = 0 and *χ*_1_ = −*χ*. Thus, the coefficients are parametrized by

17 $${f}_{0{\bf{k}}}=\,\cos \,{\theta }_{{\bf{k}}},\,{f}_{1{\bf{k}}}=\,\sin \,{\theta }_{{\bf{k}}}{{\rm{e}}}^{-i\chi }.$$

The phase *χ* parametrizes the U(1) transformation allowed by the BCS thermal vacuum, and we will show its consequence later.

The BCS thermal vacuum can be obtained by a unitary transformation of the two-mode BCS ground state. Explicitly,

18 $$|0(\beta )\rangle ={e}^{Q}|g,\tilde{g}\rangle =\prod _{{\bf{k}}}\,(\cos \,{\theta }_{{\bf{k}}}+\,\sin \,{\theta }_{{\bf{k}}}{{\rm{e}}}^{-i\chi }{\alpha }_{{\bf{k}}}^{\dagger }{\tilde{\alpha }}_{{\bf{k}}}^{\dagger })\,(\cos \,{\theta }_{{\bf{k}}}+\,\sin \,{\theta }_{{\bf{k}}}{{\rm{e}}}^{-i\chi }{\beta }_{-{\bf{k}}}^{\dagger }{\tilde{\beta }}_{-{\bf{k}}}^{\dagger })|g,\tilde{g}\rangle ,$$

where

19 $$Q=\sum _{{\bf{k}}}\,{\theta }_{{\bf{k}}}({\alpha }_{{\bf{k}}}{\tilde{\alpha }}_{{\bf{k}}}{e}^{i\chi }+{\alpha }_{{\bf{k}}}^{\dagger }{\tilde{\alpha }}_{{\bf{k}}}^{\dagger }{{\rm{e}}}^{-i\chi }+{\beta }_{-{\bf{k}}}{\tilde{\beta }}_{-{\bf{k}}}{{\rm{e}}}^{i\chi }+{\beta }_{-{\bf{k}}}^{\dagger }{\tilde{\beta }}_{-{\bf{k}}}^{\dagger }{{\rm{e}}}^{-i\chi }).$$

Following a similarity transformation using the unitary operator e^−*Q*^, the BCS thermal vacuum is the Fock-space vacuum of the thermal quasi-quanta

20 $${\alpha }_{{\bf{k}}}(T)={{\rm{e}}}^{Q}{\alpha }_{{\bf{k}}}{{\rm{e}}}^{-Q}={\alpha }_{{\bf{k}}}\,\cos \,{\theta }_{{\bf{k}}}-{\tilde{\alpha }}_{{\bf{k}}}^{\dagger }\,\sin \,{\theta }_{{\bf{k}}}{{\rm{e}}}^{-i\chi },\,{\beta }_{-{\bf{k}}}(T)={{\rm{e}}}^{Q}{\beta }_{{\bf{k}}}{{\rm{e}}}^{-Q}={\beta }_{-{\bf{k}}}\,\cos \,{\theta }_{{\bf{k}}}-{\tilde{\beta }}_{{\bf{k}}}^{\dagger }\,\sin \,{\theta }_{{\bf{k}}}{e}^{-i\chi }.$$

This is because ${\alpha }_{{\rm{k}}}(T)|0(\beta )\rangle ={{\rm{e}}}^{Q}{\alpha }_{{\rm{k}}}|g,\tilde{g}\rangle =0$ and ${\beta }_{-{\rm{k}}}(T)|0(\beta )\rangle ={{\rm{e}}}^{Q}{\beta }_{-{\rm{k}}}|g,\tilde{g}\rangle =0$. One can construct similar relations for the tilde operators. Moreover, |0(*β*)〉 is the ground state of the thermal BCS Hamiltonian

21 $${H}_{{\rm{BCS}}}(T)\equiv {{\rm{e}}}^{Q}{H}_{{\rm{BCS}}}{{\rm{e}}}^{-Q}=\sum _{{\bf{k}}}\,{E}_{{\rm{k}}}[{\alpha }_{{\bf{k}}}^{\dagger }(T){\alpha }_{{\bf{k}}}(T)+{\beta }_{-{\bf{k}}}^{\dagger }(T){\beta }_{-{\bf{k}}}(T)]+\sum _{{\bf{k}}}\,({\xi }_{{\bf{k}}}-{E}_{{\bf{k}}})+\frac{|{\rm{\Delta }}{|}^{2}}{g}.$$

Incidentally, the similarity transformation does not change the eigenvalues.

At zero temperature, | *f*_0**k**_| = 1 and *f*_1**k**_ = 0. Hence, the BCS thermal vacuum reduces to the ground state of the conventional BCS theory, but in the augmented two-mode form. It is important to notice that the two-mode BCS ground state differs from the BCS thermal vacuum at finite temperatures in their structures: The former is the Fock-space vacuum of the quasi-particles, i.e., ${\alpha }_{{\bf{k}}}|g,\tilde{g}\rangle ={\beta }_{{\bf{k}}}|g,\tilde{g}\rangle =0$; the latter is the Fock-space vacuum of the thermal quasi-particles, i.e., *α*_**k**_(*T*)|0(*β*)〉 = *β*_**k**_(*T*)|0(*β*)〉 = 0. We mention that the BCS thermal vacuum is a generalized coherent squeezed state (see the Supplemental Information for details.)

By construction, the statistical average of any physical observable can be obtained by taking the expectation value with respect to the thermal vacuum. In the following we show how this procedure reproduces the BCS number and gap equations. One can show that

22 $$\langle 0(\beta )|{\alpha }_{{\bf{k}}}^{\dagger }{\alpha }_{{\bf{k}}}|0(\beta )\rangle =\langle g,\tilde{g}|{{\rm{e}}}^{-Q}{\alpha }_{{\bf{k}}}^{\dagger }{\alpha }_{{\bf{k}}}{{\rm{e}}}^{Q}|g,\tilde{g}\rangle =f({E}_{{\bf{k}}}).$$

Here we have used Eq. (20) and ${\sin }^{2}\,{\theta }_{{\bf{k}}}=f({E}_{{\bf{k}}})$. Similarly, one can show that $\langle 0(\beta )|{\beta }_{-{\bf{k}}}^{\dagger }{\beta }_{-{\bf{k}}}|0(\beta )\rangle =f({E}_{{\bf{k}}})$, $\langle 0(\beta )|{\beta }_{-{\bf{k}}}{\beta }_{-{\bf{k}}}^{\dagger }|0(\beta )\rangle =\langle 0(\beta )|{\alpha }_{{\bf{k}}}{\alpha }_{{\bf{k}}}^{\dagger }|0(\beta )\rangle =1-f({E}_{{\bf{k}}})$, and $\langle 0(\beta )|{\beta }_{-{\bf{k}}}{\alpha }_{{\bf{k}}}|0(\beta )\rangle =\langle 0(\beta )|{\alpha }_{{\bf{k}}}^{\dagger }{\beta }_{-{\bf{k}}}^{\dagger }|0(\beta )\rangle =0$. Applying these identities and the inverse transformation of Eq. (10), the total particle number is given by the expectation value of the number operator with respect to the state |0(*β*)〉:

23 $$\begin{array}{rcl}N & = & \sum _{{\bf{k}}}\,(\langle \mathrm{0(}\beta )|{\psi }_{{\bf{k}}\uparrow }^{\dagger }{\psi }_{{\bf{k}}\uparrow }\mathrm{|0(}\beta )\rangle +\langle \mathrm{0(}\beta )|{\psi }_{-{\bf{k}}\downarrow }^{\dagger }{\psi }_{-{\bf{k}}\downarrow }\mathrm{|0(}\beta )\rangle )\\  & = & \sum _{{\bf{k}}}\,[\langle \mathrm{0(}\beta )|(|{u}_{{\bf{k}}}{|}^{2}{\alpha }_{{\bf{k}}}^{\dagger }{\alpha }_{{\bf{k}}}+|{v}_{{\bf{k}}}{|}^{2}{\beta }_{-{\bf{k}}}{\beta }_{-{\bf{k}}}^{\dagger }+\,{v}_{{\bf{k}}}{u}_{{\bf{k}}}{\beta }_{-{\bf{k}}}{\alpha }_{{\bf{k}}}+{u}_{{\bf{k}}}^{\ast }{v}_{{\bf{k}}}^{\ast }{\alpha }_{{\bf{k}}}^{\dagger }{\beta }_{-{\bf{k}}}^{\dagger }\mathrm{)|0(}\beta )\rangle \\  &  & +\,\langle \mathrm{0(}\beta )|(|{u}_{{\bf{k}}}{|}^{2}{\beta }_{-{\bf{k}}}^{\dagger }{\beta }_{-{\bf{k}}}+|{v}_{{\bf{k}}}{|}^{2}{\alpha }_{{\bf{k}}}{\alpha }_{{\bf{k}}}^{\dagger }-\,{v}_{{\bf{k}}}{u}_{{\bf{k}}}{\alpha }_{{\bf{k}}}{\beta }_{-{\bf{k}}}-{u}_{{\bf{k}}}^{\ast }{v}_{{\bf{k}}}^{\ast }{\beta }_{-{\bf{k}}}^{\dagger }{\alpha }_{{\bf{k}}}^{\dagger }\mathrm{)|0(}\beta )\rangle ]\\  & = & 2\,\sum _{{\bf{k}}}\,\{|{u}_{{\bf{k}}}{|}^{2}f({E}_{{\bf{k}}})+|{v}_{{\bf{k}}}{|}^{2}\mathrm{[1}-f({E}_{{\bf{k}}}\mathrm{)]\}.}\end{array}$$

Here $|{u}_{{\bf{k}}}{|}^{2},|{v}_{{\bf{k}}}{|}^{2}=(1\pm {\xi }_{{\bf{k}}}/{E}_{{\bf{k}}})/2$, and the expression is the same as the one from the finite-temperature Green’s function formalism^2^. The gap equation can be deduced in a similar way. Explicitly,

24 $${\rm{\Delta }}=-\,g\,\sum _{{\bf{k}}}\,\langle 0(\beta )|{\psi }_{{\bf{k}}\uparrow }{\psi }_{-{\bf{k}}\downarrow }|0(\beta )\rangle =g{\rm{\Delta }}\,\sum _{{\bf{k}}}\,\frac{1-2f({E}_{{\bf{k}}})}{2{E}_{{\bf{k}}}}.$$

When compared to the Green’s function approach^2^, the thermal-vacuum approach is formally at the quantum mechanical level. To emphasize this feature, we will use some techniques from quantum mechanics to perform calculations instead of using the framework of quantum field theory.

### Perturbation Theory Based on BCS Thermal Vacuum

The BCS equations of state can be derived from a formalism formally identical to quantum mechanics by the BCS thermal vacuum. We generalize the procedure to more complicated calculations such as evaluating higher-order corrections to the BCS mean-field theory by developing a perturbation theory like the one in quantum mechanics. The idea is to take the BCS thermal vacuum as the unperturbed state and follow the standard time-independent perturbation formalism^17^ to build the corrections order by order. To develop a perturbation theory at the quantum mechanical level based on the BCS thermal vacuum, we first identify the omitted interaction term in the BCS approximation as the perturbation. By comparing the total Hamiltonian (7) and the BCS Hamiltonian (8), one finds

25 $$H={H}_{{\rm{BCS}}}-g\,\sum _{{\bf{k}}\ne 0}\,\sum _{{\bf{p}}{\bf{q}}}\,{\psi }_{{\bf{k}}-{\bf{p}}\uparrow }^{\dagger }{\psi }_{{\bf{p}}\downarrow }^{\dagger }{\psi }_{{\bf{k}}-{\bf{q}}\downarrow }{\psi }_{{\bf{q}}\uparrow }={H}_{0}+V,$$

where the second term can be thought of as a perturbation to the BCS Hamiltonian if we take *H*_0_ ≡ *H*_BCS_ as the unperturbed Hamiltonian.

Next, the BCS thermal vacuum is used to find the contributions from the perturbation. The BCS thermal vacuum |0(*β*)〉 is the ground state of the unperturbed thermal BCS Hamiltonian *H*_BCS_(*T*) = e^*Q*^*H*_BCS_e^−*Q*^. Our task is to find the thermal vacuum |0(*β*)〉_c_ of the total thermal Hamiltonian

26 $$H(T)={{\rm{e}}}^{{Q}_{{\rm{c}}}}{H}_{{\rm{BCS}}}{{\rm{e}}}^{-{Q}_{{\rm{c}}}}+{{\rm{e}}}^{{Q}_{{\rm{c}}}}V{{\rm{e}}}^{-{Q}_{{\rm{c}}}}.$$

Here the generator becomes *Q*_c_ = *Q* + *δQ* due to the presence of the interaction. The perturbed thermal vacuum |0(*β*)〉_c_ should satisfy ${}_{{\rm{c}}}\langle 0(\beta )|{\mathscr{A}}|0(\beta )\rangle _{{\rm{c}}}=\frac{1}{Z}{\rm{Tr}}({{\rm{e}}}^{-\beta H}{\mathscr{A}})$. However, the inclusion of *V* invalidates the Bogoliubov transformation and *H* is not diagonalized by the quasi-particles *α*_**k**_ and *β*_−**k**_. As a consequence, ${}_{{\rm{c}}}\langle 0(\beta )|{\alpha }_{{\bf{k}}}^{\dagger }{\alpha }_{{\bf{k}}}|0(\beta )\rangle _{{\rm{c}}}\ne f({E}_{{\bf{k}}})$, ${}_{{\rm{c}}}\langle 0(\beta )|{\beta }_{{\bf{k}}}^{\dagger }{\beta }_{{\bf{k}}}|0(\beta )\rangle _{{\rm{c}}}\ne f({E}_{{\bf{k}}})$ and the evaluation of |0(*β*)〉_c_ can be difficult. Formally, the perturbed thermal vacuum may be expressed as $|0(\beta ){\rangle }_{{\rm{c}}}={{\rm{e}}}^{{Q}_{{\rm{c}}}}|g,\tilde{g}{\rangle }_{{\rm{c}}}$, where $|g,\tilde{g}{\rangle }_{{\rm{c}}}$ denotes the perturbed two-mode ground state. Since the thermal Hamiltonian (26) involves *Q*_c_, a full treatment of the perturbed thermal vacuum would be quite challenging.

Here we adopt the approximation ${Q}_{{\rm{c}}}\simeq Q$ in the thermal Hamiltonian (26) and obtain the corresponding thermal vacuum by a perturbation theory. When *V* = 0, |0(*β*)〉 is the ground state of the unperturbed thermal BCS Hamiltonian *H*_BCS_(*T*). We will take it as the starting point and evaluate |0(*β*)〉_c_ by applying the quantum perturbation theory^17^. Explicitly, the perturbed thermal vacuum is approximated by

27 $$|0(\beta ){\rangle }_{{\rm{c}}}\approx |0(\beta )\rangle +\sum _{k\ne 0}\,|{k}^{(0)}\rangle \frac{{V}_{k0}}{{E}_{0}^{(0)}-{E}_{k}^{(0)}}+\cdots ,$$

where *V*_*k*0_ = 〈*k*^(0)^|*V*(*T*)|0(*β*)〉 is the matrix element of the perturbation with respect to the unperturbed states, *V*(*T*) = *e*^*Q*^*Ve*^−*Q*^, |*k*^(0)^〉 includes all possible unperturbed excited states given by ${\alpha }_{{\bf{k}}}^{\dagger }(T)|0(\beta )\rangle$, ${\beta }_{-{\bf{k}}}^{\dagger }(T)|0(\beta )\rangle$, ${\beta }_{-{\bf{k}}}^{\dagger }(T){\alpha }_{{\bf{k}}}^{\dagger }(T)|0(\beta )\rangle$, etc., and ${E}_{n}^{\mathrm{(0)}}$ is the corresponding unperturbed energy. After obtaining the perturbed BCS thermal vacuum order by order, the corrections to physical quantities such as the order parameter can be found by taking the expectation values of the corresponding operators with respect to the perturbed thermal vacuum. We caution that the thermal vacuum (27) is the ground state of the thermal Hamiltonian with the unperturbed *Q* in Eq. (26).

The perturbation theory requires the evaluation of the matrix elements of the perturbation, *V*_*k*0_, $k=1,2,\ldots$, which are determined as follows. Let $|{k}^{(0)}\rangle ={{\mathscr{O}}}_{k}^{\dagger }(T)\cdots {{\mathscr{O}}}_{1}^{\dagger }(T)|0(\beta )\rangle$ be a *k*-particle excited state, where ${{\mathscr{O}}}_{i}$ represents the quasi-particle annihilation operator ${\alpha }_{{{\bf{k}}}_{i}}$ or ${\beta }_{-{{\bf{k}}}_{i}}$. Then,

28 $$\begin{array}{rcl}{V}_{k0} & = & \langle {k}^{(0)}|V(T)|0(\beta )\rangle \\  & = & \langle g,\tilde{g}|{{\rm{e}}}^{-Q}{{\mathscr{O}}}_{1}(T){{\rm{e}}}^{Q}{{\rm{e}}}^{-Q}\cdots {{\rm{e}}}^{Q}{{\rm{e}}}^{-Q}{{\mathscr{O}}}_{n}(T){{\rm{e}}}^{Q}{{\rm{e}}}^{-Q}{{\rm{e}}}^{Q}V{{\rm{e}}}^{-Q}{{\rm{e}}}^{Q}|g,\tilde{g}\rangle \\  & = & \langle g,\tilde{g}|{{\mathscr{O}}}_{1}\cdots {{\mathscr{O}}}_{n}V|g,\tilde{g}\rangle .\end{array}$$

Since the perturbation *V* is quartic in the fermion fields, the expectation values of *V*(*T*) between the thermal vacuum and odd-number excited states vanish. Therefore, 〈0(*β*)|*α*_**k**_(*T*)*V*(*T*)|0(*β*)〉 = 〈0(*β*)|*β*_−**k**_(*T*)*V*(*T*)|0(*β*)〉 = 0, and accordingly *V*_10_ = 0.

The matrix element associated with the two-particle excited states, *V*_20_, can be evaluated with the method shown in the Supplemental Information. Thus, the nonvanishing elements involve one *α*- and one *β*- quanta and are given by

29 $${V}_{20,{{\bf{l}}}_{1}{{\bf{l}}}_{2}}=-\,2g{u}_{{{\bf{l}}}_{1}}^{\ast }{v}_{{{\bf{l}}}_{1}}^{\ast }{\delta }_{{{\bf{l}}}_{1},{{\bf{l}}}_{2}}\,\sum _{q\ne {{\bf{l}}}_{1}}\,{v}_{{\bf{q}}}{v}_{{\bf{q}}}^{\ast }.$$

To evaluate the matrix element associated with the four-particle excited states, *V*_40_, we need to consider the following matrix elements

30 $$\begin{array}{c}\langle \mathrm{0(}\beta )|{\beta }_{-{{\bf{l}}}_{4}}(T){\beta }_{-{{\bf{l}}}_{3}}(T){\beta }_{-{{\bf{l}}}_{2}}(T){\beta }_{-{{\bf{l}}}_{1}}(T)V(T\mathrm{)|0(}\beta )\rangle ,\\ \langle \mathrm{0(}\beta )|{\beta }_{-{{\bf{l}}}_{4}}(T){\beta }_{-{{\bf{l}}}_{3}}(T){\beta }_{-{{\bf{l}}}_{2}}(T){\alpha }_{{{\bf{l}}}_{1}}(T)V(T\mathrm{)|0(}\beta )\rangle ,\\ \langle \mathrm{0(}\beta )|{\beta }_{-{{\bf{l}}}_{4}}(T){\beta }_{-{{\bf{l}}}_{3}}(T){\alpha }_{{{\bf{l}}}_{2}}(T){\alpha }_{{{\bf{l}}}_{1}}(T)V(T\mathrm{)|0(}\beta )\rangle ,\\ \langle \mathrm{0(}\beta )|{\beta }_{-{{\bf{l}}}_{4}}(T){\alpha }_{{{\bf{l}}}_{3}}(T){\alpha }_{{{\bf{l}}}_{2}}(T){\alpha }_{{{\bf{l}}}_{1}}(T)V(T\mathrm{)|0(}\beta )\rangle ,\\ \langle \mathrm{0(}\beta )|{\alpha }_{{{\bf{l}}}_{4}}(T){\alpha }_{{{\bf{l}}}_{3}}(T){\alpha }_{{{\bf{l}}}_{2}}(T){\alpha }_{{{\bf{l}}}_{1}}(T)V(T\mathrm{)|0(}\beta )\rangle \mathrm{.}\end{array}$$

It can be shown that only the term $\langle 0(\beta )|{\beta }_{-{{\bf{l}}}_{4}}(T){\beta }_{-{{\bf{l}}}_{3}}(T){\alpha }_{{{\bf{l}}}_{2}}(T){\alpha }_{{{\bf{l}}}_{1}}(T)V(T)|0(\beta )\rangle$ is nonzero (see the SI). Therefore, the nonvanishing matrix element is

31 $$\begin{array}{rcl}{V}_{\mathrm{40,}{{\bf{l}}}_{1}{{\bf{l}}}_{2}{{\bf{l}}}_{3}{{\bf{l}}}_{4}} & = & -g({u}_{{{\bf{l}}}_{2}}^{\ast }{u}_{{{\bf{l}}}_{4}}^{\ast }{v}_{{{\bf{l}}}_{1}}^{\ast }{v}_{{{\bf{l}}}_{3}}^{\ast }+{u}_{{{\bf{l}}}_{1}}^{\ast }{u}_{{{\bf{l}}}_{3}}^{\ast }{v}_{{{\bf{l}}}_{2}}^{\ast }{v}_{{{\bf{l}}}_{4}}^{\ast })\,\mathrm{(1}-{\delta }_{{{\bf{l}}}_{1},{{\bf{l}}}_{3}})\,\mathrm{(1}-{\delta }_{{{\bf{l}}}_{2},{{\bf{l}}}_{4}}){\delta }_{{{\bf{l}}}_{1}+{{\bf{l}}}_{2},{{\bf{l}}}_{3}+{{\bf{l}}}_{4}}\\  &  & +\,g({u}_{{{\bf{l}}}_{1}}^{\ast }{u}_{{{\bf{l}}}_{4}}^{\ast }{v}_{{{\bf{l}}}_{2}}^{\ast }{v}_{{{\bf{l}}}_{3}}^{\ast }+{u}_{{{\bf{l}}}_{2}}^{\ast }{u}_{{{\bf{l}}}_{3}}^{\ast }{v}_{{{\bf{l}}}_{1}}^{\ast }{v}_{{{\bf{l}}}_{4}}^{\ast })\,\mathrm{(1}-{\delta }_{{{\bf{l}}}_{1},{{\bf{l}}}_{4}})\,\mathrm{(1}-{\delta }_{{{\bf{l}}}_{2},{{\bf{l}}}_{3}}){\delta }_{{{\bf{l}}}_{1}+{{\bf{l}}}_{2},{{\bf{l}}}_{3}+{{\bf{l}}}_{4}}\mathrm{.}\end{array}$$

Finally, since the perturbation *V* contains at most four quasi-particle operators, all matrix elements associated with higher order (*k* > 4) excited states vanish (see Eq. (28)).

According to Eq. (27), the perturbed BCS thermal vacuum up to the first order is given by

32 $$\begin{array}{rcl}\mathrm{|0(}\beta ){\rangle }_{{\rm{c}}} & \approx  & \mathrm{|0(}\beta )\rangle +\sum _{{{\bf{l}}}_{1},{{\bf{l}}}_{2}}\,{\alpha }_{{{\bf{l}}}_{1}}^{\dagger }(T){\beta }_{-{{\bf{l}}}_{2}}^{\dagger }(T\mathrm{)|0(}\beta )\rangle \frac{{V}_{\mathrm{20,}{{\bf{l}}}_{1}{{\bf{l}}}_{2}}}{{E}_{0}^{\mathrm{(0)}}-{E}_{\mathrm{2,}{{\bf{l}}}_{1}{{\bf{l}}}_{2}}^{\mathrm{(0)}}}\\  &  & +\,\sum _{{{\bf{l}}}_{1},{{\bf{l}}}_{2},{{\bf{l}}}_{3},{{\bf{l}}}_{4}}\,{\alpha }_{{{\bf{l}}}_{1}}^{\dagger }(T){\alpha }_{{{\bf{l}}}_{2}}^{\dagger }(T){\beta }_{-{{\bf{l}}}_{3}}^{\dagger }(T){\beta }_{-{{\bf{l}}}_{4}}^{\dagger }(T\mathrm{)|0(}\beta )\rangle \frac{{V}_{\mathrm{40,}{{\bf{l}}}_{1}{{\bf{l}}}_{2}{{\bf{l}}}_{3}{{\bf{l}}}_{4}}}{{E}_{0}^{\mathrm{(0)}}-{E}_{\mathrm{4,}{{\bf{l}}}_{1}{{\bf{l}}}_{2}{{\bf{l}}}_{4}{{\bf{l}}}_{4}}^{\mathrm{(0)}}}\mathrm{.}\end{array}$$

Here the matrix elements *V*_20_ and *V*_40_ are given by Eqs (29) and (31), respectively, ${E}_{0}^{(0)}={\sum }_{{\bf{k}}}\,({\xi }_{{\bf{k}}}-{E}_{{\bf{k}}})+\frac{|{\rm{\Delta }}{|}^{2}}{g}$ is the unperturbed BCS ground-state energy, ${E}_{2,{{\bf{l}}}_{1}{{\bf{l}}}_{2}}^{(0)}={E}_{0}^{(0)}+{E}_{{{\bf{l}}}_{1}}+{E}_{{{\bf{l}}}_{2}}$ with ${E}_{{{\bf{l}}}_{1}}=\sqrt{{\xi }_{{{\bf{l}}}_{1}}^{2}+|{\rm{\Delta }}{|}^{2}}$ is the second-order excited state energy, and ${E}_{4,{{\bf{l}}}_{1}{{\bf{l}}}_{2}{{\bf{l}}}_{3}{{\bf{l}}}_{4}}^{(0)}={E}_{0}^{(0)}+{E}_{{{\bf{l}}}_{1}}+{E}_{{{\bf{l}}}_{2}}+{E}_{{{\bf{l}}}_{3}}+{E}_{{{\bf{l}}}_{4}}$ is the fourth-order excited state energy.

After obtaining the expansion of of the full BCS thermal vacuum, one can derive the corrections to the chemical potential and gap function from the expectation values of the density and pairing operators. Explicitly,

33 $$N=\sum _{{\bf{k}},\sigma =\uparrow ,\downarrow }\,{}_{{\rm{c}}}\langle \mathrm{0(}\beta )|{\psi }_{{\bf{k}}\sigma }^{\dagger }{\psi }_{{\bf{k}}\sigma }\mathrm{|0(}\beta )\rangle _{{\rm{c}}},\,{{\rm{\Delta }}}_{{\rm{c}}}=-\,g\,\sum _{{\bf{k}}}\,{}_{{\rm{c}}}\langle \mathrm{0(}\beta )|{\psi }_{{\bf{k}}\uparrow }{\psi }_{-{\bf{k}}\downarrow }\mathrm{|0(}\beta )\rangle _{{\rm{c}}}\mathrm{.}$$

It can be shown that the terms associated with *V*_40_ do not contribute to *N* or Δ_c_. Following the convention of the BCS theory, we assume that the order parameter is a real number. We summarize the derivations in the SI and present here the final expressions. The number equation becomes

34 $$N=\sum _{{\bf{k}}}\,[1-\frac{{\xi }_{{\bf{k}}}}{{E}_{{\bf{k}}}}+2\frac{{\xi }_{{\bf{k}}}}{{E}_{{\bf{k}}}}f({E}_{{\bf{k}}})]+\sum _{{\bf{k}}}\,\frac{1-f({E}_{{\bf{k}}})}{2{E}_{{\bf{k}}}^{2}}\frac{g{{\rm{\Delta }}}^{2}}{{E}_{0}^{\mathrm{(0)}}-{E}_{\mathrm{2,}{\bf{k}}{\bf{k}}}^{\mathrm{(0)}}}\,\sum _{{\bf{q}}\ne {\bf{k}}}\,(1-\frac{{\xi }_{{\bf{q}}}}{{E}_{{\bf{q}}}})+O({V}^{2}\mathrm{).}$$

By solving the full chemical potential *μ*_*c*_ from the equation, one can obtain the correction to *μ*. The expansion of the order parameter can be obtained in a similar fashion:

35 $${{\rm{\Delta }}}_{{\rm{c}}}={\rm{\Delta }}-{g}^{2}\,\sum _{{\bf{k}}}\,\frac{1-f({E}_{{\bf{k}}})}{8{E}_{{\bf{k}}}^{3}}\frac{{{\rm{\Delta }}}^{2}{\xi }_{{\bf{k}}}}{{E}_{{\bf{k}}}}\,\sum _{{\bf{q}}\ne {\bf{k}}}\,(1-\frac{{\xi }_{{\bf{q}}}}{{E}_{{\bf{q}}}})+O({V}^{2}\mathrm{).}$$

Here we emphasize that Δ_c_ (Δ) denotes the full (unperturbed) order parameter.

The first-order correction to the order parameter can be found numerically for different coupling strengths. The fermion density is $n=\frac{N}{{\mathscr{V}}}=\frac{{k}_{F}^{3}}{3{\pi }^{2}}$ with *k*_*F*_ being the Fermi momentum of a noninteracting Fermi gas with the same particle density. The Fermi energy is defined by ${E}_{F}={\hslash }^{2}{k}_{F}^{2}/(2m)$. We first solve the unperturbed equations of states, Eqs (23) and (24), at different temperatures to obtain Δ and *μ*. Then, we substitute the unperturbed solution to Eq. (35) and get the first-order correction to Δ_c_(*T*). The critical temperature *T*_*c*_ can be found by checking where the full order parameter Δ_c_(*T*) vanishes. Eq. (35) indicates the full order parameter is lowered by the first-order correction. We found that the critical temperature *T*_*c*_ is also lowered when compared to the unperturbed value. However, our numerical results show that the correction to the critical temperature is small if the particle-particle interaction is weak. The strong correction to *T*_*c*_ due to the particle-hole channel (induced interaction)^13,14^ is not included in the BCS theory. Since the perturbation *V* considered here carries non-zero momentum, the calculation here shows the correction from finite-momentum effects to the Cooper pairs.

**Figure 1:**
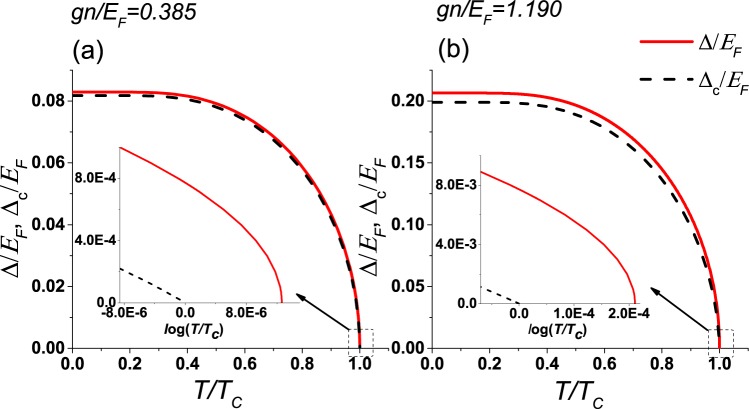
The unperturbed (red solid lines) and perturbed (black dashed lines) order parameters as functions of temperature for (**a**) *gn*/*E*_*F*_ = 0.385 and (**b**) *gn*/*E*_*F*_ = 1.19. The perturbation is truncated at the second order. The insets show the details of the curves near *T*_*c*_, and there the temperature is shown in logarithmic scale. Here ${E}_{F}={\hslash }^{2}{k}_{F}^{2}/(2m)$ is the Fermi energy of a noninteracting Fermi gas with the same density.

Figure 1 shows the unperturbed and perturbed (up to the first-order correction) order parameters as functions of temperature. In Fig. 1(a), a relatively small coupling constant *gn*/*E*_*F*_ = 0.385 is chosen, which corresponds to the conventional BCS case where the interaction energy is much smaller compared to the Fermi energy. We found $\delta {\rm{\Delta }}/{\rm{\Delta }}\simeq {10}^{-2}$ at any temperature below *T*_*c*_ (which is determined by Δ_c_(*T*_*c*_) = 0), where *δ*Δ = Δ_c_ − Δ is the first order correction of the order parameter. Hence, *δ*Δ is indeed small in the BCS limit. We also found the ratio $\frac{{{\rm{\Delta }}}_{{\rm{c}}}(0)}{{k}_{B}{T}_{c}}\simeq 1.72$, which is close to the mean-field BCS result of 1.76^2,23^. In Fig. 1(b), a relatively large coupling constant *gn*/*E*_*F*_ = 1.19 is chosen. The first-order correction *δ*Δ/Δ is more visible, but the ratio is still less than 8% at any temperature below *T*_*c*_. We also found $\frac{{{\rm{\Delta }}}_{{\rm{c}}}(0)}{{k}_{B}{T}_{c}}\simeq 1.61$, which is more distinct from the unperturbed BCS value. According to the value of Δ(*T* = 0)/*E*_*F*_ indicated by Fig. 1, the system with *gn*/*E*_*F*_ = 0.385 is in the BCS regime while the one with *gn*/*E*_*F*_ = 1.19 is beyond the BCS limit because of its relatively large gap. The perturbation calculations allow us to improve the results order by order, but the complexity of the calculations increases rapidly. The insets of Fig. 1 show the detailed behavior close to *T*_*c*_ and indeed the critical temperature is lowered by the perturbation.

We remark that the BCS theory is usually viewed as a variational theory^23,24,27^. The introduction of the perturbation calculation using the BCS thermal vacuum reproduces the thermodynamics of the BCS theory at the lowest order, and it introduces a quantum-mechanical style perturbation theory. The corrections to the BCS theory thus can be obtained by the perturbation theory, albeit the exact results require a full treatment of the transformation from the two-mode vacuum to the thermal vacuum.

### Pairing Correlation

In principle, the thermal vacuum provides a method for directly evaluating the statistical (thermal) average of any operator constructed from the fermion field *ψ*. Here we demonstrate another example by analyzing the correlation between the Cooper pairs, coming from higher moments of the order parameter. We introduce the pairing correlation

36 $${{\rm{\Delta }}}_{{\rm{p}}}^{2}={\langle {V}_{p}\rangle }^{2}-\langle {V}_{p}^{2}\rangle ,\,{\rm{where}}\,{V}_{p}=g\,\sum _{{\bf{p}}}\,{\psi }_{-{\bf{p}}\downarrow }{\psi }_{{\bf{p}}\uparrow }$$

is the pairing operator and 〈*V*_*p*_〉 = Δ (or Δ_c_ depending on whether the unperturbed or perturbed BCS thermal vacuum is used) if we choose the order parameter to be real. If the system obeys number conservation or the Wick decomposition^25^, then Δ^2^*p* = 0 and the system exhibits no pairing correlation. A straightforward calculation shows that Δ^2^*p* ≥ 0 at the level of the unperturbed BCS thermal vacuum:

37 $${{\rm{\Delta }}}^{2}-{\langle {V}_{p}^{2}\rangle }_{0}={{\rm{\Delta }}}^{2}-\langle \mathrm{0(}\beta )|{V}_{p}^{2}\mathrm{|0(}\beta )\rangle ={g}^{2}\,\sum _{{\bf{p}}}\,{\{\frac{{\rm{\Delta }}}{2{E}_{{\bf{p}}}}\mathrm{[1}-2f({E}_{{\bf{p}}})]\}}^{2}\mathrm{.}$$

We remark that one may consider the pairing fluctuation by defining ${\tilde{{\rm{\Delta }}}}_{{\rm{p}}}^{2}=\langle {V}_{p}^{\dagger }{V}_{p}\rangle -|\langle {V}_{p}\rangle {|}^{2}$. However, this expression mixes both the pair-pair (e.g. ${\psi }^{\dagger }{\psi }^{\dagger }$ or *ψψ*) correlation as well as the density-density (e.g. ${\psi }^{\dagger }\psi$) correlation and does not clearly reveal the effects from pairing. Therefore, we use the expression (36) to investigate the pair-pair correlation.

Next, we use the perturbation theory to evaluate higher-order corrections to the pairing correlation. We take the perturbative BCS thermal vacuum shown in Eq. (32) and estimate the correction to the pairing correlation:

38 $$\begin{array}{rcl}{{\rm{\Delta }}}_{{\rm{p}}}^{2} & = & {{\rm{\Delta }}}_{{\rm{c}}}^{2}-{}_{{\rm{c}}}\langle \mathrm{0(}\beta )|{V}_{p}^{2}\mathrm{|0(}\beta )\rangle _{{\rm{c}}}\\  & = & -\,{g}^{2}\,\sum _{{\bf{p}}}\,{({u}_{{\bf{p}}}{v}_{{\bf{p}}}{\sin }^{2}{\theta }_{{\bf{p}}}-{u}_{{\bf{p}}}{v}_{{\bf{p}}}{\cos }^{2}{\theta }_{{\bf{p}}})}^{2}+\sum _{{{\bf{l}}}_{1}}\,\mathrm{[2}{g}^{2}{u}_{{{\bf{l}}}_{1}}^{2}\,{\cos }^{2}\,{\theta }_{{{\bf{l}}}_{1}}{\rm{\Delta }}\\  &  & -\,2{g}^{3}{u}_{{{\bf{l}}}_{1}}^{3}{v}_{{{\bf{l}}}_{1}}\,{\cos }^{2}\,{\theta }_{{{\bf{l}}}_{1}}\,\cos \,2{\theta }_{{{\bf{l}}}_{1}}]\frac{{u}_{{{\bf{l}}}_{1}}{v}_{{{\bf{l}}}_{1}}\,{\sum }_{{\bf{q}}\ne {{\bf{l}}}_{1}}\,{v}_{{\bf{q}}}{v}_{{\bf{q}}}}{{E}_{{{\bf{l}}}_{1}}}\\  &  & +\,{g}^{2}{(\sum _{{\bf{p}}}{u}_{{\bf{p}}}{v}_{{\bf{p}}}\cos 2{\theta }_{{\bf{p}}})}^{2}-\sum _{{{\bf{l}}}_{1}}\,\mathrm{[2}{g}^{2}{v}_{{{\bf{l}}}_{1}}^{2}\,{\cos }^{2}\,{\theta }_{{{\bf{l}}}_{1}}{\rm{\Delta }}\\  &  & -\,2{g}^{3}{u}_{{{\bf{l}}}_{1}}{v}_{{{\bf{l}}}_{1}}^{3}\,{\cos }^{2}\,{\theta }_{{{\bf{l}}}_{1}}\,\cos \,2{\theta }_{{{\bf{l}}}_{1}}]\frac{{u}_{{{\bf{l}}}_{1}}{v}_{{{\bf{l}}}_{1}}\,{\sum }_{{\bf{q}}\ne {{\bf{l}}}_{1}}\,{v}_{{\bf{q}}}{v}_{{\bf{q}}}}{{E}_{{{\bf{l}}}_{1}}}\\  &  & +\,\sum _{{{\bf{l}}}_{1}\ne {{\bf{l}}}_{2}}\,4{g}^{3}{u}_{{{\bf{l}}}_{1}}^{2}{u}_{{{\bf{l}}}_{2}}^{2}\,{\cos }^{2}\,{\theta }_{{{\bf{l}}}_{1}}\,{\cos }^{2}\,{\theta }_{{{\bf{l}}}_{2}}\frac{{u}_{{{\bf{l}}}_{2}}{v}_{{{\bf{l}}}_{2}}{u}_{{{\bf{l}}}_{1}}{v}_{{{\bf{l}}}_{1}}}{({E}_{{{\bf{l}}}_{1}}+{E}_{{{\bf{l}}}_{2}})}\\  &  & +\,\sum _{{{\bf{l}}}_{1}\ne {{\bf{l}}}_{2}}\,4{g}^{3}{v}_{{{\bf{l}}}_{1}}^{2}{v}_{{{\bf{l}}}_{2}}^{2}\,{\cos }^{2}\,{\theta }_{{{\bf{l}}}_{1}}\,{\cos }^{2}\,{\theta }_{{{\bf{l}}}_{2}}\frac{{u}_{{{\bf{l}}}_{2}}{v}_{{{\bf{l}}}_{2}}{u}_{{{\bf{l}}}_{1}}{v}_{{{\bf{l}}}_{1}}}{({E}_{{{\bf{l}}}_{1}}+{E}_{{{\bf{l}}}_{2}})}-{{\rm{\Delta }}}_{{\rm{c}}}^{2}\mathrm{.}\end{array}$$

The expression of ${}_{{\rm{c}}}\langle \mathrm{0(}\beta )|{V}_{p}^{2}\mathrm{|0(}\beta )\rangle _{{\rm{c}}}$ to the first order is shown in the Supplemental Information.

**Figure 2:**
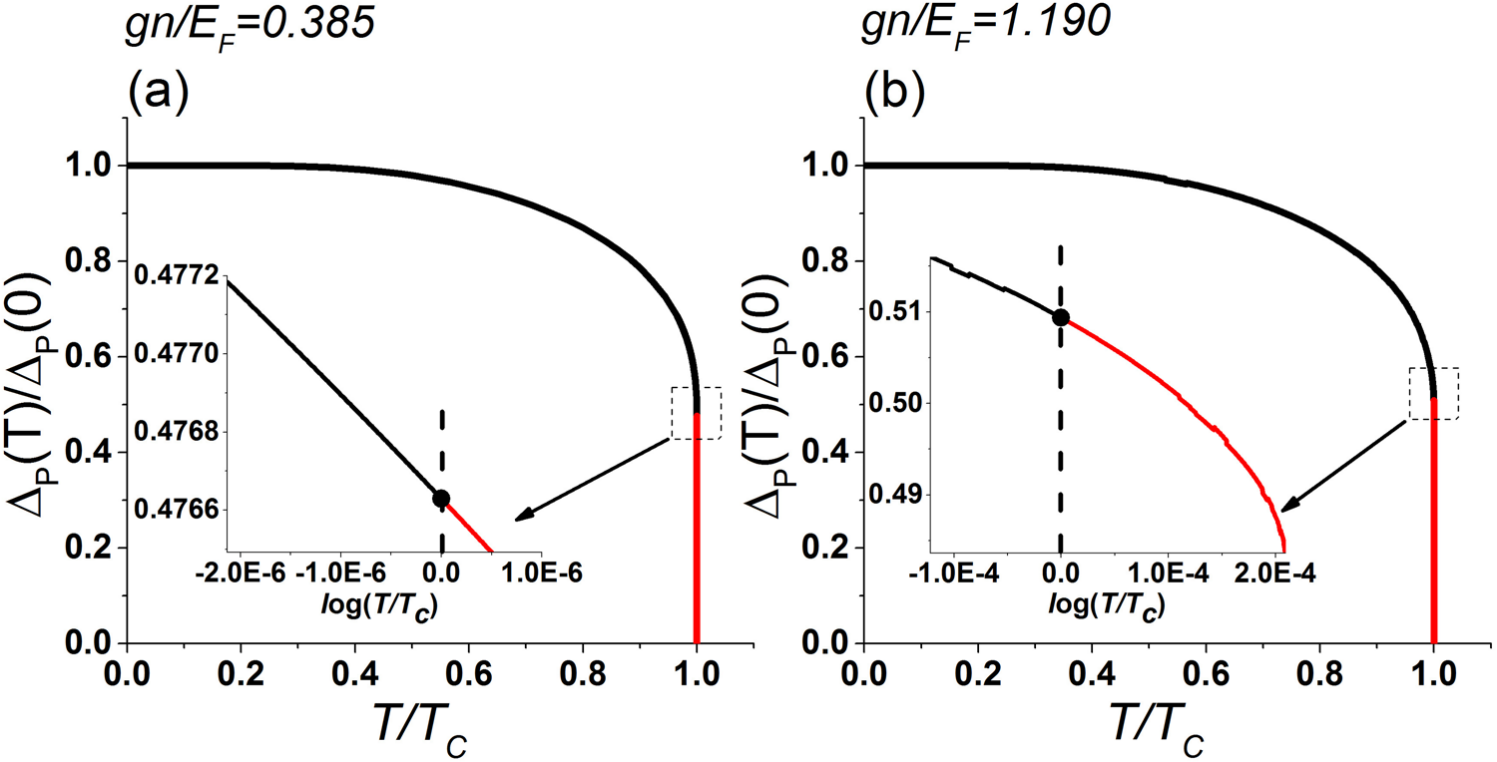
The pairing correlation Δ_p_(*T*), up to the first order according to Eq. (38), as a function of temperature (normalized by Δ_p_(*T* = 0)) for (**a**) *gn*/*E*_*F*_ = 0.385 and (**b**) *gn*/*E*_*F*_ = 1.19, respectively. The insets show the behavior close to *T*_*c*_ (indicated by the black dots). The curves below and above *T*_*c*_ are colored by black and red to emphasize the pseudogap effect, where the pairing correlation persists above *T*_*c*_.

Figure 2 shows the pairing fluctuation Δ_p_ up to the first order according to Eq. (38) as functions of temperature. The pairing fluctuation decreases with temperature but increases with the interaction. By examining Δ_p_ near *T*_*c*_, we found that the pairing correlation survives in a small region above the corrected *T*_*c*_. In other words, when the corrected order parameter Δ_*c*_ vanishes, the pairing correlation persists. This may be considered as evidence of the pseudogap effect^19,20^, where pairing effects still influence the system above *T*_*c*_. We mention that in the Ginzburg-Landau theory, the Ginzburg criterion checks the fluctuation of the specific heat^28^ to identify the critical regime. Here we directly evaluate the pairing correlation by applying the BCS thermal vacuum and its perturbation theory by estimating the correction from the original fermion-fermion interaction. Our results provide a direct calculation of the pairing correlation and offer support for the pseudogap phenomenon.

### Geometric Phase of BCS Thermal Vacuum

In the derivation of the unperturbed BCS thermal vacuum, we introduced a phase *χ* (see Eq. (18)). Since |0(*β*)〉 may be considered as a pure quantum state, the system can acquire a geometric phase similar to the Berry phase^21^ when *χ* evolves adiabatically along a closed loop *C* in the parameter space. We call it the thermal phase *γ*(*C*), which can be evaluated as follows.

39 $$\gamma (C)=i\,{\oint }_{C}\,dt\langle \mathrm{0(}\beta ),\chi (t)|\frac{d}{dt}|\mathrm{0(}\beta ),\chi (t)\rangle =i\,{\int }_{0}^{2\pi }\,d\chi \langle \mathrm{0(}\beta ),\chi |\frac{\partial }{\partial \chi }|\mathrm{0(}\beta ),\chi \rangle \mathrm{.}$$

By using the Baker-Campbell-Hausdorff disentangling formula^29,30^, the thermal vacuum can be written as

40 $$\mathrm{|0(}\beta )\rangle ={{\rm{e}}}^{{\sum }_{{\bf{k}}}2\mathrm{ln}\cos {\theta }_{{\bf{k}}}}{{\rm{e}}}^{{\sum }_{{\bf{k}}}\tan {\theta }_{{\bf{k}}}{{\rm{e}}}^{-i\chi }({\alpha }_{{\bf{k}}}^{\dagger }{\tilde{\alpha }}_{{\bf{k}}}^{\dagger }+{\beta }_{-{\bf{k}}}^{\dagger }{\tilde{\beta }}_{-{\bf{k}}}^{\dagger })}|g,\tilde{g}\rangle \mathrm{.}$$

After some algebra, we obtain $i\langle 0(\beta )|\frac{\partial }{\partial \chi }|0(\beta )\rangle =2\,{\sum }_{{\bf{k}}}\,f({E}_{{\bf{k}}})$. Therefore, the thermal phase is

41 $$\gamma (C)=4\pi \,\sum _{{\bf{k}}}\,f({E}_{{\bf{k}}})=2\pi [{N}_{\alpha }(\beta )+{N}_{\beta }(\beta )],$$

which is proportional to the total quasi-particle number *N*_*α*_(*β*) + *N*_*β*_(*β*) at temperature $T=\frac{1}{{k}_{B}\beta }$. The quasi-particle number is nonzero only when *T* > 0.

The origin of the thermal phase can be understood as follows. The thermal vacuum can be thought of as a purification of the mixed state at finite temperatures. This can be clarified by noting that Eq. (5) leads to

42 $$\mathrm{|0(}\beta )\rangle =\sum _{n}\,\frac{1}{\sqrt{Z}}{{\rm{e}}}^{-\frac{\beta {E}_{n}}{2}-in\chi }|n,\tilde{n}\rangle =\sqrt{{\rho }_{\beta }}\,\sum _{n}\,{\mathscr{U}}|n,\tilde{n}\rangle ,$$

where ${\rho }_{\beta }=\frac{1}{Z}{{\rm{e}}}^{-\beta H}$ is the density matrix of the non-tilde system at temperature *T*, and the phase *nχ* is because each excitation of a quasi-particle contributes e^−*iχ*^ indicated by Eq. (18). The unitary operator ${\mathscr{U}}$ has the following matrix representation in the basis formed by {|*n*〉}:

43 $$(\begin{array}{cccc}1 & 0 & 0 & \cdots \\ 0 & {e}^{-i\chi } & 0 & \cdots \\ 0 & 0 & {e}^{-2i\chi } & \cdots \\ \vdots  & \vdots  & \vdots  & \ddots \end{array})\mathrm{.}$$

There is another way of purifying the density matrix^16,31^ by defining the amplitude of the density matrix as

44 $${w}_{\beta }=\sqrt{{\rho }_{\beta }}{\mathscr{U}}=\sum _{n}\,\frac{1}{\sqrt{Z}}{{\rm{e}}}^{-\frac{\beta H}{2}}|n\rangle \langle n|{{\rm{e}}}^{-in\chi }.$$

By comparing Eqs (42) and (44), one can find a one-to-one mapping between the thermal vacuum |0(*β*)〉 and the amplitude *w*_*β*_.

However, the purification of a mixed state is not unique. For instance, Eq. (17) allows a relative phase *χ* between *f*_0**k**_ and *f*_1**k**_. A U(1) transformation corresponding to a change of the parameter *χ* leads to another thermal vacuum. Hence, the BCS thermal vacuum can be parametrized in the space 0 ≤ *χ* < 2*π*, i.e. one may recognize the collection of BCS thermal vacua as a U(1) manifold parametrized by *χ*. The thermal phase (41) from the BCS thermal vacuum may be understood as follows. If *χ* is transported along the U(1) manifold along a loop, every excited quasi-particle acquires a phase 2*π*. Therefore, the thermal phase indicates the number of thermal excitations in the system.

When *T* → 0, the statistical average becomes the expectation value with respect to the ground state. In the present case, the BCS thermal vacuum reduces to the (two-mode) BCS ground state. As a consequence, the U(1) manifold of the unitary transformation of the BCS thermal vacua is no longer defined at *T* = 0 because there is no thermal excitation and *f*_1**k**_ = 0 in Eq. (16). Importantly, the BCS ground state is already a pure state at *T* = 0, so there is no need to introduce *χ* for parametrizing the manifold of the unitary transformation in the purification. Hence, the thermal phase should only be defined at finite temperatures when the system is thermal.

## Discussion

By introducing the two-mode BCS vacuum and the corresponding unitary transformation, we have shown how to construct the BCS thermal vacuum. A perturbation theory is then developed based on the BCS thermal vacuum. In principle, one can evaluate the corrections from the original fermion interactions ignored in the BCS approximation. Importantly, the perturbation calculations are at the quantum-mechanical level even though the BCS theory and the BCS thermal vacuum are based on quantum field theory. The interaction, however, renders the transformation from the two-mode ground state to the thermal vacuum nonlinear. We took an approximation in developing the perturbation theory, and a full treatment awaits future investigations.

The BCS thermal vacuum is expected to offer insights into interacting quantum many-body systems. We have shown that the pairing correlation from the perturbation theory persists when the corrected order parameter vanishes, providing evidence of the pseudogap phenomenon. The thermal phases associated with the BCS thermal vacuum elucidates the internal geometry of its construction. The BCS thermal vacuum and its perturbation theory thus offers an alternative way for investigating superconductivity and superfluidity.

### Data availability

All data generated or analysed during this study are included in this published article (and its Supplementary Information files).

## Electronic supplementary material

Supplemental Information
