# Supplementary material for: BCS thermal vacuum of fermionic superfluids and its perturbation theory
Source: Sci Rep. 2018 Aug 10;8:11995. doi: 10.1038/s41598-018-30438-1 (PMC6086907; doi:10.1038/s41598-018-30438-1)
Supplement: Supplementary file 1 — Supplemental Information [file 41598_2018_30438_MOESM1_ESM.pdf]

Supplemental Information: BCS thermal vacuum of fermionic superfluids and its perturbation theory

Xu-Yang Hou,[1] Ziwen Huang,[1] Hao Guo,[1,*] Yan He,[2] and Chih-Chun Chien[3,†]

[1]Department of Physics, Southeast University, Jiulonghu Campus, Nanjing 211189, China
[2]College of Physical Science and Technology, Sichuan University, Chengdu, Sichuan 610064, China
[3]School of Natural Sciences, University of California, Merced, CA 95343, USA

I. DETAILS OF PERTURBATION THEORY BASED ON BCS THERMAL VACUUM

Here are some details of the calculations involving the BCS thermal vacuum. For the calculations of the matrix elements $V_{20,\mathbf{l}_1\mathbf{l}_2}$ in the perturbation theory, we evaluate

$$\begin{aligned}
&\langle 0(\beta)|\beta_{-\mathbf{l}_2}(T)\alpha_{\mathbf{l}_1}(T)V(T)|0(\beta)\rangle = -g\sum_{\mathbf{k}\neq 0}\sum_{\mathbf{pq}}\langle g,\tilde{g}|\mathrm{e}^{-Q}\beta_{-\mathbf{l}_2}(T)\alpha_{\mathbf{l}_1}(T)\mathrm{e}^{Q}(u^*_{\mathbf{k-p}}\alpha^\dagger_{\mathbf{k-p}}+v_{\mathbf{k-p}}\beta_{\mathbf{p-k}})\\
\times&\ (u^*_{-\mathbf{p}}\beta^\dagger_{\mathbf{p}}-v_{-\mathbf{p}}\alpha_{-\mathbf{p}})(u_{\mathbf{q-k}}\beta_{\mathbf{k-q}}-v^*_{\mathbf{q-k}}\alpha^\dagger_{\mathbf{q-k}})(u_{\mathbf{q}}\alpha_{\mathbf{q}}+v^*_{\mathbf{q}}\beta^\dagger_{-\mathbf{q}})\mathrm{e}^{-Q}\mathrm{e}^{Q}|g,\tilde{g}\rangle\\
=&\ -g\sum_{\mathbf{k}\neq 0}\sum_{\mathbf{pq}}(u^*_{\mathbf{k-p}}u^*_{-\mathbf{p}}u_{\mathbf{q-k}}v^*_{\mathbf{q}}\delta_{\mathbf{l}_1,\mathbf{k-p}}\delta_{-\mathbf{l}_2,\mathbf{p}}\delta_{\mathbf{k-q},-\mathbf{q}}\\
+&\ u^*_{\mathbf{k-p}}v_{-\mathbf{p}}v^*_{\mathbf{q-k}}v^*_{\mathbf{q}}\delta_{-\mathbf{p},\mathbf{q-k}}\delta_{\mathbf{l}_1,\mathbf{k-p}}\delta_{-\mathbf{l}_2,-\mathbf{q}}-v_{\mathbf{k-p}}u^*_{-\mathbf{p}}v^*_{\mathbf{q-k}}v^*_{\mathbf{q}}\delta_{\mathbf{l}_1,\mathbf{q-k}}\delta_{\mathbf{p-k},\mathbf{p}}\delta_{-\mathbf{l}_2,-\mathbf{q}}\\
+&\ v_{\mathbf{k-p}}u^*_{-\mathbf{p}}v^*_{\mathbf{q-k}}v^*_{\mathbf{q}}\delta_{\mathbf{l}_1,\mathbf{q-k}}\delta_{-\mathbf{l}_2,\mathbf{p}}\delta_{\mathbf{p-k},-\mathbf{q}})\\
=&\ -2gu^*_{\mathbf{l}_1}v^*_{\mathbf{l}_1}\delta_{\mathbf{l}_1,\mathbf{l}_2}\sum_{\mathbf{q}\neq\mathbf{l}_1}v_{\mathbf{q}}v^*_{\mathbf{q}},
\end{aligned}\tag{1}$$

where we have used $0=\alpha_{\mathbf{k}}|g,\tilde{g}\rangle=\langle g,\tilde{g}|\alpha^\dagger_{\mathbf{k}}=\beta_{-\mathbf{k}}|g,\tilde{g}\rangle=\langle g,\tilde{g}|\beta^\dagger_{-\mathbf{k}}$, and $|u_{\mathbf{k}}|^2,|v_{\mathbf{k}}|^2=(1\pm\xi_{\mathbf{k}}/E_{\mathbf{k}})/2$. Similar calculations lead to $\langle 0(\beta)|\alpha_{\mathbf{l}_2}(T)\alpha_{\mathbf{l}_1}(T)V(T)|0(\beta)\rangle=0=\langle 0(\beta)|\beta_{-\mathbf{l}_2}(T)\beta_{-\mathbf{l}_1}(T)V(T)|0(\beta)\rangle$. Therefore, $V_{20,\mathbf{l}_1\mathbf{l}_2}=V_{20,\mathbf{l}_1}\delta_{\mathbf{l}_1,\mathbf{l}_2}$ with $V_{20,\mathbf{l}_1}=-2gu^*_{\mathbf{l}_1}v^*_{\mathbf{l}_1}\sum_{\mathbf{q}\neq\mathbf{l}_1}v_{\mathbf{q}}v^*_{\mathbf{q}}$.

Next, we evaluate $V_{40,\mathbf{l}_1\mathbf{l}_2\mathbf{l}_3\mathbf{l}_4}$:

$$\begin{aligned}
\langle 0(\beta)|\beta_{-\mathbf{l}_4}(T)\beta_{-\mathbf{l}_3}(T)\alpha_{\mathbf{l}_2}(T)\alpha_{\mathbf{l}_1}(T)V(T)|0(\beta)\rangle &= -\sum_{\mathbf{k}\neq 0}\sum_{\mathbf{pq}}\langle g,\tilde{g}|\beta_{-\mathbf{l}_4}\beta_{-\mathbf{l}_3}\alpha_{\mathbf{l}_2}\alpha_{\mathbf{l}_1}V|g,\tilde{g}\rangle\\
=&-g(u^*_{\mathbf{l}_2}u^*_{\mathbf{l}_4}v^*_{\mathbf{l}_1}v^*_{\mathbf{l}_3}+u^*_{\mathbf{l}_1}u^*_{\mathbf{l}_3}v^*_{\mathbf{l}_2}v^*_{\mathbf{l}_4})(1-\delta_{\mathbf{l}_1,\mathbf{l}_3})(1-\delta_{\mathbf{l}_2,\mathbf{l}_4})\delta_{\mathbf{l}_1+\mathbf{l}_2,\mathbf{l}_3+\mathbf{l}_4}\\
&+g(u^*_{\mathbf{l}_1}u^*_{\mathbf{l}_4}v^*_{\mathbf{l}_2}v^*_{\mathbf{l}_3}+u^*_{\mathbf{l}_2}u^*_{\mathbf{l}_3}v^*_{\mathbf{l}_1}v^*_{\mathbf{l}_4})(1-\delta_{\mathbf{l}_1,\mathbf{l}_4})(1-\delta_{\mathbf{l}_2,\mathbf{l}_3})\delta_{\mathbf{l}_1+\mathbf{l}_2,\mathbf{l}_3+\mathbf{l}_4}.
\end{aligned}\tag{2}$$

Hence,

$$\begin{aligned}
V_{40,\mathbf{l}_1\mathbf{l}_2\mathbf{l}_3\mathbf{l}_4}=&-g(u^*_{\mathbf{l}_2}u^*_{\mathbf{l}_4}v^*_{\mathbf{l}_1}v^*_{\mathbf{l}_3}+u^*_{\mathbf{l}_1}u^*_{\mathbf{l}_3}v^*_{\mathbf{l}_2}v^*_{\mathbf{l}_4})(1-\delta_{\mathbf{l}_1,\mathbf{l}_3})(1-\delta_{\mathbf{l}_2,\mathbf{l}_4})\delta_{\mathbf{l}_1+\mathbf{l}_2,\mathbf{l}_3+\mathbf{l}_4}\\
&+g(u^*_{\mathbf{l}_1}u^*_{\mathbf{l}_4}v^*_{\mathbf{l}_2}v^*_{\mathbf{l}_3}+u^*_{\mathbf{l}_2}u^*_{\mathbf{l}_3}v^*_{\mathbf{l}_1}v^*_{\mathbf{l}_4})(1-\delta_{\mathbf{l}_1,\mathbf{l}_4})(1-\delta_{\mathbf{l}_2,\mathbf{l}_3})\delta_{\mathbf{l}_1+\mathbf{l}_2,\mathbf{l}_3+\mathbf{l}_4}.
\end{aligned}\tag{3}$$

The total particle number is given by the expectation value of the ψ-quantum number operator with respect to the state $|0(\beta)\rangle_{\mathrm{c}}$:

$$\begin{aligned}
N=&\sum_{\mathbf{k},\sigma=\uparrow,\downarrow}{}_{\mathrm{c}}\langle 0(\beta)|\psi^\dagger_{\mathbf{k}\sigma}\psi_{\mathbf{k}\sigma}|0(\beta)\rangle_{\mathrm{c}}=\sum_{\mathbf{k}}\langle 0(\beta)|\big(\psi^\dagger_{\mathbf{k}\uparrow}\psi_{\mathbf{k}\uparrow}+\psi^\dagger_{-\mathbf{k}\downarrow}\psi_{-\mathbf{k}\downarrow}\big)|0(\beta)\rangle\\
&+\sum_{\mathbf{l}_3}\sum_{\mathbf{l}_1,\mathbf{l}_2}\big(\langle 0(\beta)|\psi^\dagger_{\mathbf{l}_3\uparrow}\psi_{\mathbf{l}_3\uparrow}\alpha^\dagger_{\mathbf{l}_1}(T)\beta^\dagger_{-\mathbf{l}_2}(T)|0(\beta)\rangle\frac{V_{20,\mathbf{l}_1\mathbf{l}_2}}{E^{(0)}_0-E^{(0)}_{2,\mathbf{l}_1\mathbf{l}_2}}+\langle 0(\beta)|\psi^\dagger_{-\mathbf{l}_3\downarrow}\psi_{-\mathbf{l}_3\downarrow}\alpha^\dagger_{\mathbf{l}_1}(T)\beta^\dagger_{-\mathbf{l}_2}(T)|0(\beta)\rangle\frac{V_{20,\mathbf{l}_1\mathbf{l}_2}}{E^{(0)}_0-E^{(0)}_{2,\mathbf{l}_1\mathbf{l}_2}}\big)\\
&+\sum_{\mathbf{l}_3}\sum_{\mathbf{l}_1,\mathbf{l}_2}\big(\langle 0(\beta)|\beta_{-\mathbf{l}_2}(T)\alpha_{\mathbf{l}_1}(T)\psi^\dagger_{\mathbf{l}_3\uparrow}\psi_{\mathbf{l}_3\uparrow}|0(\beta)\rangle\frac{V^*_{20,\mathbf{l}_1\mathbf{l}_2}}{E^{(0)}_0-E^{(0)}_{2,\mathbf{l}_1\mathbf{l}_2}}+\langle 0(\beta)|\beta_{-\mathbf{l}_2}(T)\alpha_{\mathbf{l}_1}(T)\psi^\dagger_{-\mathbf{l}_3\downarrow}\psi_{-\mathbf{l}_3\downarrow}|0(\beta)\rangle\frac{V^*_{20,\mathbf{l}_1\mathbf{l}_2}}{E^{(0)}_0-E^{(0)}_{2,\mathbf{l}_1\mathbf{l}_2}}\big)\\
&+O(V^2)\\
&=\sum_{\mathbf{k}}\Big(1-\frac{\xi_{\mathbf{k}}}{E_{\mathbf{k}}}+2\frac{\xi_{\mathbf{k}}}{E_{\mathbf{k}}}f(E_{\mathbf{k}})\Big)+\sum_{\mathbf{k}}\frac{1-f(E_{\mathbf{k}})}{E_{\mathbf{k}}}\frac{\Delta V_{20,\mathbf{k}}+\Delta^*V^*_{20,\mathbf{k}}}{E^{(0)}_0-E^{(0)}_{2,\mathbf{kk}}}+O(V^2).
\end{aligned}\tag{4}$$

The perturbation series of the gap function can be derived in a similar fashion.

To evaluate the pairing correlation, we calculate the unperturbed expectation

$$\begin{aligned}\langle 0(\beta)|V_p^2|0(\beta)\rangle &= g^2\sum_{\mathbf{pq}}\langle g,\tilde{g}|\mathrm{e}^{-Q}(u_{\mathbf{p}}\alpha_{\mathbf{p}}+v^*_{\mathbf{p}}\beta^\dagger_{-\mathbf{p}})(u_{\mathbf{p}}\beta_{-\mathbf{p}}-v^*_{\mathbf{p}}\alpha^\dagger_{\mathbf{p}})(u_{\mathbf{q}}\alpha_{-\mathbf{q}}+v^*_{\mathbf{q}}\beta^\dagger_{\mathbf{q}})(u_{\mathbf{q}}\beta_{\mathbf{q}}-v^*_{\mathbf{q}}\alpha^\dagger_{-\mathbf{q}})\mathrm{e}^{Q}|g,\tilde{g}\rangle \\ &= -g^2\sum_{\mathbf{p}}(u_{\mathbf{p}}v^*_{\mathbf{p}}\sin^2\theta_{\mathbf{p}}-u_{\mathbf{p}}v^*_{\mathbf{p}}\cos^2\theta_{\mathbf{p}})^2+g^2\big(\sum_{\mathbf{p}}u^*_{\mathbf{p}}v_{\mathbf{p}}\cos 2\theta_{\mathbf{p}}\big)^2. \qquad (5)\end{aligned}$$

By using the BCS thermal vacuum, we obtain the following expression to the first order.

$$\begin{aligned}\langle V_p^2\rangle &\equiv {}_{\mathrm{c}}\langle 0(\beta)|V_p^2|0(\beta)\rangle_{\mathrm{c}} \\ &= \langle 0(\beta)|V_p^2|0(\beta)\rangle \\ &+\sum_{\mathbf{l}_1,\mathbf{l}_2}\big(\langle 0(\beta)|V_p^2\alpha^\dagger_{\mathbf{l}_1}(T)\beta^\dagger_{-\mathbf{l}_2}(T)|0(\beta)\rangle\frac{V_{20,\mathbf{l}_1\mathbf{l}_2}}{E_0^{(0)}-E^{(0)}_{2,\mathbf{l}_1\mathbf{l}_2}}+\sum_{\mathbf{l}_1,\mathbf{l}_2}\big(\langle 0(\beta)|\beta_{-\mathbf{l}_2}(T)\alpha_{\mathbf{l}_1}(T)V_p^2|0(\beta)\rangle\frac{V^*_{20,\mathbf{l}_1\mathbf{l}_2}}{E_0^{(0)}-E^{(0)}_{2,\mathbf{l}_1\mathbf{l}_2}} \\ &+\sum_{\mathbf{l}_1,\mathbf{l}_2,\mathbf{l}_3,\mathbf{l}_4}\frac{V_{40,\mathbf{l}_1\mathbf{l}_2\mathbf{l}_3\mathbf{l}_4}}{E_0^{(0)}-E^{(0)}_{4,\mathbf{l}_1\mathbf{l}_2\mathbf{l}_3\mathbf{l}_4}}\big(\langle 0(\beta)|V_p^2\alpha^\dagger_{\mathbf{l}_1}(T)\alpha^\dagger_{\mathbf{l}_2}(T)\beta^\dagger_{-\mathbf{l}_3}(T)\beta^\dagger_{-\mathbf{l}_4}(T)|0(\beta)\rangle \\ &+\sum_{\mathbf{l}_1,\mathbf{l}_2,\mathbf{l}_3,\mathbf{l}_4}\frac{V^*_{40,\mathbf{l}_1\mathbf{l}_2\mathbf{l}_3\mathbf{l}_4}}{E_0^{(0)}-E^{(0)}_{4,\mathbf{l}_1\mathbf{l}_2\mathbf{l}_3\mathbf{l}_4}}\big(\langle 0(\beta)|\beta_{-\mathbf{l}_4}(T)\beta_{-\mathbf{l}_3}(T)\alpha_{\mathbf{l}_2}(T)\alpha_{\mathbf{l}_1}(T)V_p^2|0(\beta)\rangle. \\ &= \langle V_p^2\rangle_0+\sum_{\mathbf{l}_1,\mathbf{l}_2}\Big(\frac{V_{p2,\mathbf{l}_1\mathbf{l}_2}V_{20,\mathbf{l}_1\mathbf{l}_2}}{E_0^{(0)}-E^{(0)}_{2,\mathbf{l}_1\mathbf{l}_2}}+\frac{V^*_{p2,\mathbf{l}_1\mathbf{l}_2}V^*_{20,\mathbf{l}_1\mathbf{l}_2}}{E_0^{(0)}-E^{(0)}_{2,\mathbf{l}_1\mathbf{l}_2}}\Big)+\sum_{\mathbf{l}_1,\mathbf{l}_2,\mathbf{l}_3,\mathbf{l}_4}\Big(\frac{V_{p4,\mathbf{l}_1\mathbf{l}_2\mathbf{l}_3\mathbf{l}_4}V_{40,\mathbf{l}_1\mathbf{l}_2\mathbf{l}_3\mathbf{l}_4}}{E_0^{(0)}-E^{(0)}_{4,\mathbf{l}_1\mathbf{l}_2\mathbf{l}_3\mathbf{l}_4}}+\frac{V^*_{p4,\mathbf{l}_1\mathbf{l}_2\mathbf{l}_3\mathbf{l}_4}V^*_{40,\mathbf{l}_1\mathbf{l}_2\mathbf{l}_3\mathbf{l}_4}}{E_0^{(0)}-E^{(0)}_{4,\mathbf{l}_1\mathbf{l}_2\mathbf{l}_3\mathbf{l}_4}}\Big), \qquad (6)\end{aligned}$$

where

$$\begin{aligned}&V_{p2,\mathbf{l}_1\mathbf{l}_2}=\langle 0(\beta)|V_p^2\alpha^\dagger_{\mathbf{l}_1}(T)\beta^\dagger_{-\mathbf{l}_2}(T)|0(\beta)\rangle,\quad V^*_{p2,\mathbf{l}_1\mathbf{l}_2}=\langle 0(\beta)|\beta_{-\mathbf{l}_2}(T)\alpha_{\mathbf{l}_1}(T)(V_p^\dagger)^2|0(\beta)\rangle, \\ &V_{p4,\mathbf{l}_1\mathbf{l}_2\mathbf{l}_3\mathbf{l}_4}=\langle 0(\beta)|V_p^2\alpha^\dagger_{\mathbf{l}_1}(T)\alpha^\dagger_{\mathbf{l}_2}(T)\beta^\dagger_{-\mathbf{l}_3}(T)\beta^\dagger_{-\mathbf{l}_4}(T)|0(\beta)\rangle,\ V^*_{p4,\mathbf{l}_1\mathbf{l}_2\mathbf{l}_3\mathbf{l}_4}=\langle 0(\beta)|\beta_{-\mathbf{l}_4}(T)\beta_{-\mathbf{l}_3}(T)\alpha_{\mathbf{l}_2}(T)\alpha_{\mathbf{l}_1}(T)(V_p^\dagger)^2|0(\beta)\rangle. \\ &\qquad (7)\end{aligned}$$

We remark that $V_p^\dagger\neq V_p$. The coefficients V_{p2} and V_{p4} are evaluated as follows.

$$\begin{aligned}V_{p2,\mathbf{l}_1\mathbf{l}_2} &= [2gu^2_{\mathbf{l}_1}\cos^2\theta_{\mathbf{l}_1}\Delta^*-2g^2u^3_{\mathbf{l}_1}v^*_{\mathbf{l}_1}\cos^2\theta_{\mathbf{l}_1}\cos 2\theta_{\mathbf{l}_1}]\delta_{\mathbf{l}_1,\mathbf{l}_2}, &(8)\\ V^*_{p2,\mathbf{l}_1\mathbf{l}_2} &= [-2gv^{*2}_{\mathbf{l}_1}\cos^2\theta_{\mathbf{l}_1}\Delta^*+2g^2u_{\mathbf{l}_1}v^{*3}_{\mathbf{l}_1}\cos^2\theta_{\mathbf{l}_1}\cos 2\theta_{\mathbf{l}_1}]\delta_{\mathbf{l}_1,\mathbf{l}_2}, &(9)\\ V_{p4,\mathbf{l}_1\mathbf{l}_2\mathbf{l}_3\mathbf{l}_4} &= -2g^2u^2_{\mathbf{l}_1}u^2_{\mathbf{l}_2}\cos^2\theta_{\mathbf{l}_1}\cos^2\theta_{\mathbf{l}_2}(\delta_{\mathbf{l}_1,\mathbf{l}_3}\delta_{\mathbf{l}_2,\mathbf{l}_4}-\delta_{\mathbf{l}_1,\mathbf{l}_4}\delta_{\mathbf{l}_2,\mathbf{l}_3}), &(10)\\ V^*_{p4,\mathbf{l}_1\mathbf{l}_2\mathbf{l}_3\mathbf{l}_4} &= -2g^2v^{*2}_{\mathbf{l}_1}v^{*2}_{\mathbf{l}_2}\cos^2\theta_{\mathbf{l}_1}\cos^2\theta_{\mathbf{l}_2}(\delta_{\mathbf{l}_1,\mathbf{l}_3}\delta_{\mathbf{l}_2,\mathbf{l}_4}-\delta_{\mathbf{l}_1,\mathbf{l}_4}\delta_{\mathbf{l}_2,\mathbf{l}_3}). &(11)\end{aligned}$$

Therefore, Eq. (6) becomes

$$\begin{aligned}\langle V_p^2\rangle &= \langle V_p^2\rangle_0+\sum_{\mathbf{l}_1}[2g^2u^2_{\mathbf{l}_1}\cos^2\theta_{\mathbf{l}_1}\Delta^*-2g^3u^3_{\mathbf{l}_1}v^*_{\mathbf{l}_1}\cos^2\theta_{\mathbf{l}_1}\cos 2\theta_{\mathbf{l}_1}]\frac{u^*_{\mathbf{l}_1}v^*_{\mathbf{l}_1}\sum_{\mathbf{q}\neq\mathbf{l}_1}v_{\mathbf{q}}v^*_{\mathbf{q}}}{E_{\mathbf{l}_1}} \\ &- \sum_{\mathbf{l}_1}[2g^2v^{*2}_{\mathbf{l}_1}\cos^2\theta_{\mathbf{l}_1}\Delta^*-2g^3u_{\mathbf{l}_1}v^{*3}_{\mathbf{l}_1}\cos^2\theta_{\mathbf{l}_1}\cos 2\theta_{\mathbf{l}_1}]\frac{u_{\mathbf{l}_1}v_{\mathbf{l}_1}\sum_{\mathbf{q}\neq\mathbf{l}_1}v^*_{\mathbf{q}}v_{\mathbf{q}}}{E_{\mathbf{l}_1}} \\ &+ \sum_{\mathbf{l}_1\neq\mathbf{l}_2}4g^3u^2_{\mathbf{l}_1}u^2_{\mathbf{l}_2}\cos^2\theta_{\mathbf{l}_1}\cos^2\theta_{\mathbf{l}_2}\frac{u^*_{\mathbf{l}_2}v^*_{\mathbf{l}_2}u^*_{\mathbf{l}_1}v^*_{\mathbf{l}_1}}{(E_{\mathbf{l}_1}+E_{\mathbf{l}_2})}+\sum_{\mathbf{l}_1\neq\mathbf{l}_2}4g^3v^{*2}_{\mathbf{l}_1}v^{*2}_{\mathbf{l}_2}\cos^2\theta_{\mathbf{l}_1}\cos^2\theta_{\mathbf{l}_2}\frac{u_{\mathbf{l}_2}v_{\mathbf{l}_2}u_{\mathbf{l}_1}v_{\mathbf{l}_1}}{(E_{\mathbf{l}_1}+E_{\mathbf{l}_2})}, \qquad (12)\end{aligned}$$

where $\langle V_p^2\rangle_0$ is given by Eq.(5).

II. BCS THERMAL VACUUM IS A GENERALIZED SQUEEZED COHERENT STATE

The unperturbed BCS thermal vacuum itself has some interesting properties. Since the BCS thermal vacuum is obtained from a Bogoliubov transformation, it is a generalized coherent state. To verify the conjecture, we follow Ref.[1] and introduce the temperature-independent spin operators

$$S^+_{\alpha\mathbf{k}}=\alpha^\dagger_{\mathbf{k}}\tilde{\alpha}^\dagger_{\mathbf{k}},\quad S^-_{\alpha\mathbf{k}}=(S^+_{\alpha\mathbf{k}})^\dagger=\tilde{\alpha}_{\mathbf{k}}\alpha_{\mathbf{k}},\quad S^z_{\alpha\mathbf{k}}=\frac{1}{2}[S^+_{\alpha\mathbf{k}},S^-_{\alpha\mathbf{k}}]=\frac{1}{2}(\alpha^\dagger_{\mathbf{k}}\alpha_{\mathbf{k}}+\tilde{\alpha}^\dagger_{\mathbf{k}}\tilde{\alpha}_{\mathbf{k}}-1),$$

$$S^+_{\beta\mathbf{k}}=\beta^\dagger_{-\mathbf{k}}\tilde{\beta}^\dagger_{-\mathbf{k}},\quad S^-_{\beta\mathbf{k}}=(S^+_{\beta\mathbf{k}})^\dagger=\tilde{\beta}_{-\mathbf{k}}\beta_{-\mathbf{k}},\quad S^z_{\beta\mathbf{k}}=\frac{1}{2}[S^+_{\beta\mathbf{k}},S^-_{\beta\mathbf{k}}]=\frac{1}{2}(\beta^\dagger_{-\mathbf{k}}\beta_{-\mathbf{k}}+\tilde{\beta}^\dagger_{-\mathbf{k}}\tilde{\beta}_{-\mathbf{k}}-1), \tag{13}$$

which satisfy the SU(2) algebra since $[S^z_{\alpha\mathbf{k}},S^\pm_{\alpha\mathbf{k}}]=\pm S^\pm_{\alpha\mathbf{k}}$ and $[S^z_{\beta\mathbf{k}},S^\pm_{\beta\mathbf{k}}]=\pm S^\pm_{\beta\mathbf{k}}$. The BCS thermal vacuum can be rewritten as

$$|0(\beta)\rangle=\mathrm{e}^{\sum_\mathbf{k}(\theta_\mathbf{k}\mathrm{e}^{-i\chi}S^+_{\alpha\mathbf{k}}-\theta_\mathbf{k}\mathrm{e}^{i\chi}S^-_{\alpha\mathbf{k}})}\mathrm{e}^{\sum_\mathbf{k}(\theta_\mathbf{k}\mathrm{e}^{-i\chi}S^+_{\beta\mathbf{k}}-\theta_\mathbf{k}\mathrm{e}^{i\chi}S^-_{\beta\mathbf{k}})}|g,\tilde{g}\rangle. \tag{14}$$

Since $S^-_{\alpha\mathbf{k}}|g,\tilde{g}\rangle=S^-_{\beta\mathbf{k}}|g,\tilde{g}\rangle=0$, the above expression shows that the BCS thermal vacuum is indeed a generalized SU(2) coherent state[1]. However, it has another important property: The BCS thermal vacuum is a nilpotent coherent state because $(S^+_{\beta\mathbf{k}})^2=0=(S^-_{\beta\mathbf{k}})^2$. The identity should be understood as an identity in the Fock space of the quasi-particles. However, it is important to notice that the BCS thermal vacuum is not a coherent state with respect to the ψ_σ-quanta.

Moreover, the BCS thermal vacuum is a squeezed state associated with $S^{x,y}_{\alpha\mathbf{k}}$, $S^{x,y}_{\beta\mathbf{k}}$. Here

$$S^x_{\alpha\mathbf{k}}=\frac{1}{2}(S^+_{\alpha\mathbf{k}}+S^-_{\alpha\mathbf{k}}),\quad S^y_{\alpha\mathbf{k}}=\frac{1}{2i}(S^+_{\alpha\mathbf{k}}-S^-_{\alpha\mathbf{k}}),\quad S^x_{\beta\mathbf{k}}=\frac{1}{2}(S^+_{\beta\mathbf{k}}+S^-_{\beta\mathbf{k}}),\quad S^y_{\beta\mathbf{k}}=\frac{1}{2i}(S^+_{\beta\mathbf{k}}-S^-_{\beta\mathbf{k}}). \tag{15}$$

That means the BCS thermal vacuum saturates the the Robertson-Schrodinger inequality[2]

$$\sigma_A^2\sigma_B^2\ge|\frac{1}{2}\langle\{A,B\}\rangle-\langle A\rangle\langle B\rangle|^2+|\frac{1}{2i}\langle[A,B]\rangle|^2, \tag{16}$$

where $A=S^x_{\alpha,\beta\mathbf{k}}$, $B=S^y_{\alpha,\beta\mathbf{k}}$, $\{A,B\}$ and $[A,B]$ denote the anti-commutator and commutator of A and B. The proof that the BCS thermal vacuum leads to an equal sign in the above inequality is summarized below. Therefore, the BCS thermal vacuum is a squeezed coherent state.

By applying the relation $[S^x_{\alpha,\beta\mathbf{k}},S^y_{\alpha,\beta\mathbf{k}}]=iS^z_{\alpha,\beta\mathbf{k}}$ for the temperature-independent spin operators, the proof of the saturation of the Robertson-Schrodinger inequality is equivalent to the proof of

$$(\langle S^{x2}_{\beta\mathbf{k}}\rangle-\langle S^x_{\beta\mathbf{k}}\rangle^2)(\langle S^{y2}_{\beta\mathbf{k}}\rangle-\langle S^y_{\beta\mathbf{k}}\rangle^2)=(\langle S^x_{\beta\mathbf{k}}\rangle\langle S^y_{\beta\mathbf{k}}\rangle)^2+\frac{1}{4}\langle S^z_{\beta\mathbf{k}}\rangle^2 \tag{17}$$

with respect to the BCS thermal vacuum $|0(\beta)\rangle$. With the help of Eq. (20) in the main text, we have

$$\begin{aligned}\langle S^x_{\beta\mathbf{k}}\rangle&=\frac{1}{2}\langle\beta^\dagger_{-\mathbf{k}}\tilde{\beta}^\dagger_{-\mathbf{k}}\rangle+\frac{1}{2}\langle\tilde{\beta}_{-\mathbf{k}}\beta_{-\mathbf{k}}\rangle\\&=\frac{1}{2}(\sin\theta_\mathbf{k}\cos\theta_\mathbf{k}\mathrm{e}^{i\chi}+\sin\theta_\mathbf{k}\cos\theta_\mathbf{k}\mathrm{e}^{-i\chi})\langle g,\tilde{g}|\tilde{\beta}_{-\mathbf{k}}\tilde{\beta}^\dagger_{-\mathbf{k}}|g,\tilde{g}\rangle\\&=\sin\theta_\mathbf{k}\cos\theta_\mathbf{k}\cos\chi.\end{aligned} \tag{18}$$

Similarly, the other terms are evaluated as follows

$$\langle S^y_{\beta\mathbf{k}}\rangle=\sin\theta_\mathbf{k}\cos\theta_\mathbf{k}\sin\chi,\quad\langle S^{x2}_{\beta\mathbf{k}}\rangle=\langle S^{y2}_{\beta\mathbf{k}}\rangle=\frac{1}{4},\quad\langle S^z_{\beta\mathbf{k}}\rangle=\frac{1}{2}(2\sin^2\theta_\mathbf{k}-1). \tag{19}$$

The left-hand-side of Eq. (17) is

$$(\langle S^{x2}_{\beta\mathbf{k}}\rangle-\langle S^x_{\beta\mathbf{k}}\rangle^2)(\langle S^{y2}_{\beta\mathbf{k}}\rangle-\langle S^y_{\beta\mathbf{k}}\rangle^2)=\frac{1}{16}-\frac{1}{4}\sin^2\theta_\mathbf{k}\cos^2\theta_\mathbf{k}+\sin^4\theta_\mathbf{k}\cos^4\theta_\mathbf{k}\sin^2\chi\cos^2\chi. \tag{20}$$

The right-hand-side of Eq. (17), after some algebra, also gives the same expression. Therefore, the Robertson-Schrodinger inequality is saturated by the BCS thermal vacuum. A similar derivation for the α-quanta shows the identity also holds. Hence, the BCS thermal vacuum is indeed a generalized squeezed state.

---

* Electronic address: guohao.ph@seu.edu.cn

† Electronic address: cchien5@ucmerced.edu

1 H. Guo, Y. He, and C. C. Chien, Phys. Lett. A **381**, 351 (2017).

2 E. Merzbacher, *Quantum Mechanics* (John Wiley & Sons, Hoboken, NJ, 1998), 3rd ed.
